# The Influence of Multiple Factors on Musicology Doctoral Students’ Academic Performance: An Empirical Study Based in China

**DOI:** 10.3390/bs14111073

**Published:** 2024-11-11

**Authors:** Tingyu Yan, Hong Yu, Jiajun Tang

**Affiliations:** 1 School of Music, South China Normal University, Guangzhou 510006, China; 2 School of Music, The Ohio State University, Columbus, OH 43210, USA; 3 School of Physics, South China University of Technology, Guangzhou 510641, China; jiajunta@usc.edu; 4 Rossier School of Education, University of Southern California, Los Angeles, CA 90089, USA

**Keywords:** doctoral students’ academic performance, mental health, academic anxiety, teacher support, public health

## Abstract

As doctoral education evolves globally, the focus intensifies on doctoral candidates’ academic performance and psychological well-being. Previous research has studied the effects of individual, societal, and environmental factors on students’ academic achievements. However, there is still a lack of investigation into how these factors interact, especially in the domains of arts. This study surveyed 213 Chinese musicology doctoral students and adopted Partial Least Squares Structural Equation Modeling (PLS-SEM) to evaluate how various factors affect academic and mental health outcomes. The analysis shows that factors including teacher support, student engagement, and well-being positively influence academic performance, while academic anxiety negatively impacts academic performance. Moreover, variables help diminish academic anxiety, encompassing self-efficacy, parental support, time management skills, and student engagement. Meanwhile, enhanced doctoral students’ well-being is related to robust teacher support, facilitating conditions, and active student engagement. Notably, students who experience academic anxiety about their studies generally have lower well-being. These findings indicate that alleviating academic anxiety, refining academic resources, and reinforcing mental health supports can foster academic and psychological outcomes for doctoral students. Our study contributes vital empirical data to developing higher education policies, benefiting doctoral students’ mental health and academic success.

## 1. Introduction

Doctoral programs serve as key components of a national innovation framework by preparing innovative talent necessary for scientific development [1]. The journey of earning a doctoral degree is lengthy and strenuous, pushing students to their limits (e.g., intellect, health, emotions, and finances) [2]. The process’s rigorousness causes common issues such as high dropout rates, prolonged durations to degree completion, and low student satisfaction [3]. The high dropout rates lead to huge losses of educational resources and negative impacts on doctoral candidates’ academic and professional paths. According to the Council of Graduate Schools, the non-completion rates over seven years are as high as 70% and 60% for humanities and social sciences students. The ones at ten years are 51% and 44%. A consistent pattern worldwide is that natural sciences and engineering students typically obtain their degrees within 6 to 7 years, compared to 7 to 9 years for humanities and social sciences [4]. Furthermore, a *Nature* survey in 2019 stated around 36% of doctoral students experience depression and anxiety. Those in China face even higher risks, and 40% of doctoral students suffer from depression [5].

The number of doctoral degree holders is rising in Asia (i.e., in China, India, and South Korea), reflecting the spiraling growth of higher education and research capacity in these countries [6]. Since 2018, China has led the world in the number of doctoral degrees awarded, with the second-largest doctoral student population [7]. Increasingly adopting Western models of doctoral education, these countries focus on international exposure and research skills to compete on a global scale [8]. Governments have stimulated doctoral education through policy reforms and substantial investments [9]. For instance, China rebuilt its elite universities via the “Project 985” and “Project 211” initiatives, while India made the “Researchers’ Plan” providing financial support for doctoral students, and South Korea initiated “Brain Korea 21” enhancing doctoral candidates’ research quality and international competitiveness. Collaborative programs have also been with Western institutions via student exchanges and joint training programs for better research quality and global reach [10]. Although there is more research on doctoral students’ academic performance, much focuses on those with difficulties. Less attention was paid to the factors leading to academic success. As the doctoral student population diversifies, it is critical for developing countries to identify the key factors contributing to academic success and to create more effective support strategies for their doctoral communities.

As academic research has progressed, scholars have paid more attention to the combined effects of personal, social, and environmental factors on doctoral students’ academic performance. At the individual level, it is more likely for students with high self-efficacy to actively participate in activities that drive academic success [11]. In addition, students with effective time allocation usually achieve higher grades [12]. Nonetheless, academic anxiety harms academic performance, leading to avoidance behaviors and lowering academic achievement [13]. Meanwhile, students with higher levels of well-being had better performance and lower levels of burnout [14]. Regarding social factors, teacher support is crucial to student engagement and academic achievement since teachers can provide students with the necessary emotional and pedagogical resources [15]. Similarly, parental support equips students with emotional support and motivation for their academic goals [16]. In addition, highly engaged students generally outperform others academically; they are more likely to stay focused on their studies and demonstrate resilience in the face of challenges [17]. Finally, environmental factors of accommodations, such as the availability of learning resources and supportive learning environments, enable students to realize their academic potential [18]. However, existing research remains insufficient in exploring how these factors interactively influence musicology doctoral students’ academic performance. Further research should fill the academic gap and provide significant empirical evidence for academic support measures for this specific group.

Past studies focusing on predictors of doctoral candidates’ success emphasized metrics such as academic scores, quantity of published works, rates of program completion, and graduation percentages [19]. However, these conventional academic measures do not fully encompass doctoral students’ diverse roles in fields like interdisciplinary collaboration, the development of academic networks, and expanding their scholarly influence [20]. In contrast, more stringent and broad-based benchmarks are imposed in doctoral programs for individual achievements. These criteria demand not just stellar performance in coursework but also superior abilities in teaching, critical thinking, self-guided learning, and skills in both written and oral academic communication, alongside enduring resilience [21]. Typically, these skills are enhanced through interactive engagements with mentors and peers, as well as continuous cycles of writing and receiving feedback [22]. Additionally, the structure of the curriculum and the socialization activities within academic departments are critical in fostering the development of critical thinking and self-learning capabilities, guiding students to refine their academic focus and progressively forge their scholarly identities [23]. Although there is extensive research concerning doctoral students, a noticeable gap exists in quantitative studies examining doctoral candidates’ academic performance in music.

### Current Trends in Doctoral Music Education in China

The emergence of musicology as an independent discipline dates back to late 19th-century Europe, particularly in Germany. With the trend towards academic specialization, musicology gradually separated from broader arts studies to become its field [24]. Leipzig and Berlin universities were among the first to award doctoral degrees in musicology at the start of the 20th century, marking the discipline’s formal establishment [25]. Throughout the 20th century, musicology doctoral programs expanded rapidly worldwide, particularly in the U.S. and U.K., broadening their focus from Western classical music to global traditional music, resulting in a notable rise in doctoral degrees awarded.

The rapid progression of doctoral education in musicology on a global scale has led China to develop a robust doctoral system since it started recruiting and training musicology doctoral students in 1981. This system has matured significantly over the past forty years. Presently, 14 institutions in China are authorized to enroll doctoral students in the primary discipline of music and dance. The admissions policy usually limits each professor to a maximum of two doctoral students, reflecting the stringent criteria for granting doctoral degrees. The doctoral program at the Shanghai Conservatory of Music typically takes three years to complete, although students can extend their studies for up to six years if necessary. During this period, doctoral students are required to earn 74 credit hours, publish papers in Social Sciences Citation Index (SSCI) or Chinese Social Sciences Citation Index (CSSCI) journals, consistently submit reading notes, deliver at least two academic presentations, and participate actively in specialized seminars. A mid-term evaluation critically examines their ideological, professional, and research abilities and determines whether they can advance to doctoral candidacy. To graduate, students must produce an original dissertation of at least 100,000 words and pass a final review. Identifying the factors that influence musicology doctoral students’ academic success is key to helping them overcome challenges and complete their degrees successfully.

Recently, the Chinese Ministry of Education has heightened its regulatory standards for postgraduate education, implementing a “strict entry, strict exit” policy. The 2019 “Notice on Further Standardizing and Strengthening Postgraduate Training and Management” mandates early intervention for students who may not meet required standards, stressing the importance of strengthening these processes [26]. While these measures aim to raise the academic quality of doctoral programs, improving musicology doctoral students’ academic performance in China remains a significant global challenge.

Using structural equation modeling, this study investigates the critical factors that influence the academic success of musicology doctoral students. According to previous literature, we arranged a survey of 213 musicology doctoral students, and the responses were analyzed using PLS-SEM. The findings illustrate how various factors contribute to doctoral success, mapping the interconnected pathways that drive academic achievement. Although research on doctoral student performance has increased, there are still significant gaps in understanding, particularly in musicology. This study addresses those gaps by identifying the factors that influence academic performance in musicology, thereby providing a foundation for developing solid academic support systems. Furthermore, the growing recognition of mental health problems like anxiety and depression in doctoral students, especially in the high-pressure academic environment, highlights the strong connection between mental well-being and academic performance. This research also examines the critical role of environmental and social support systems in promoting the health and academic success of doctoral students, emphasizing the need for policies that improve the educational environment for doctoral candidates. The insights gained from this study aim to enhance academic performance while contributing to the development of targeted public health and higher education policies worldwide.

## 2. Literature Review

Firstly, we list the metrics used to assess doctoral students’ academic performance. We then review existing studies of the various factors influencing their academic achievements. Drawing from this body of research, we identify four internal factors, two external support factors, and two contextual and environmental influences, providing a comprehensive evaluation of each.

### 2.1. Comprehensive Assessment Criteria for Doctoral Students’ Academic Performance and Implementation

Doctoral academic performance is shaped by various factors, emphasizing the importance of analyzing these elements to enhance doctoral programs. Academic publications are often viewed as a primary indicator of academic achievement, particularly in competitive academic settings where slight differences in yearly publication rates can have a significant impact on doctoral students’ career prospects [27]. Other critical indicators of doctoral program quality include dropout rates, completion rates, and the rates of on-time graduation. As per the standards of the European Higher Education Area (EHEA), doctoral candidates are required to (1) possess a systematic understanding of their research field and related research methods; (2) demonstrate the ability to bridge theory and practice innovatively; (3) contribute new insights to the existing knowledge base; (4) publish innovative research at national or international levels; (5) critically analyze and integrate complex ideas; (6) effectively communicate with peers, the scholarly community, and the public; (7) enhance their own academic and professional development [28]. With a multidimensional approach, this framework analyzes doctoral students’ academic performance, combines theoretical and practical perspectives, fosters innovative research, and addresses critical aspects of career development. The application of this framework facilitates detailed analysis and offers strong evidence for improving doctoral education.

### 2.2. Academic Performance-Influencing Complex Factors and Interactive Mechanisms

The literature highlights doctoral education’s complex nature, with internal and external factors interactively shaping academic success [29]. External factors encompass mentorship, personal and social conditions, departmental support, social integration processes, and financial resources, and internal factors include motivation, writing abilities, and forming an academic identity [30]. These elements are interconnected in the doctoral education system via intricate feedback mechanisms. For instance, a student’s motivation is influenced by their living circumstances, their advisors’ support, and their participation in academic networks [31]. This suggests that motivation is not solely an individual effort but is embedded in the broader academic environment. Furthermore, social integration, closely linked to external factors such as personal and social challenges, is crucial in advancing a doctoral student’s academic journey through formal and informal interactions [32]. Beyond improving writing skills, advisor support is essential for developing a solid academic identity through continuous social engagement. The factor network shows academic performance is shaped by an interrelated set of dimensions rather than isolated elements. Formal and informal interactions are vital for doctoral students’ integration into the academic community and their academic and personal development [33]. In this study, key predictors identified in the literature are examined, including academic performance, self-efficacy, advisor support, family support, time management skills, access to resources, student engagement, academic anxiety, and well-being. These factors will be thoroughly explored and analyzed in the following sections.

### 2.3. Self-Efficacy

Bandura’s concept of self-efficacy refers to an individual’s belief in their ability to successfully accomplish a specific task. Specifically, its main sources are performance accomplishments, emotional states, vicarious experiences, and social persuasion [34], which provide crucial support for doctoral students in their learning and research endeavors [35]. On the one hand, self-efficacy and both intrinsic and extrinsic motivation impact doctoral students’ academic outcomes [36]. On the other hand, they strengthen their psychological resilience [37]. Higher levels of self-efficacy improve students’ academic engagement and persistence and reduce negative emotions so that they have better academic performance [38]. Furthermore, self-efficacy is recognized as an important predictor of academic success among doctoral students [39]. Musicology doctoral students need to build confidence in academic research and maintain a strong sense of self-identity and professional confidence in various areas (e.g., artistic creation and performance) [40]. Meanwhile, they are required to show high levels of cognitive ability, critical thinking, and creativity [40]. These demanding expectations result in academic pressures and influence both their mental health and academic performance [41]. Enhanced self-efficacy increases students’ motivation and confidence in learning and contributes to improved performance in cross-cultural academic exchanges [42]. Therefore, examining the role of self-efficacy among musicology doctoral students in China, particularly its influence on mitigating academic anxiety and enhancing academic achievement, is of significant practical relevance.

### 2.4. Teacher Support

Teacher support refers to the guidance, encouragement, and assistance provided by instructors in the academic and professional development of students. Its theoretical foundation is based on social support theory [43]. This theory argues that the support individuals receive from their social networks can strengthen psychological well-being, promote happiness, and foster personal growth and development [43]. Teachers are key figures in this support system and play a crucial role in helping students achieve their academic and career goals (i.e., by offering academic advice, emotional backing, and opportunities for research collaboration) [44]. Despite the inherent complexity and uncertainty of doctoral research, teacher support helps students overcome academic challenges and strengthen their research skills and self-confidence through academic feedback, career planning, and research opportunities [45]. Research indicates that emotional support from teachers can effectively reduce anxiety and stress among doctoral students, improving their psychological well-being and overall happiness [46]. In the field of music, teachers’ artistic guidance and collaboration on projects are especially important for doctoral students, as they must manage both theoretical research and artistic creation and performance [47]. Musicology doctoral students in China often rely on their supervisors for academic and resource support in a highly competitive environment. However, cultural notions of hierarchy can impede effective communication between teachers and students, underscoring the need for active and engaged mentorship [48]. Therefore, examining the impact of teacher support on students’ academic performance and well-being not only contributes to improving doctoral training systems but also plays a vital role in fostering students’ academic and professional success.

### 2.5. Parental Support

Parental support refers to the emotional, financial, and academic assistance provided by parents, grounded in family systems theory, which highlights the family as a vital component of the social support network that plays a key role in students’ development and success. Parental support is typically divided into three categories: emotional, financial, and academic. Emotional support helps students navigate academic and social pressures through encouragement and understanding, while financial support involves covering educational and living expenses [49]. Research shows that in doctoral education, emotional support can significantly reduce academic anxiety, improve mental health, and enhance doctoral students’ academic focus [50]. Financial support helps ease economic burdens, contributing to higher academic productivity [51]. However, excessive parental involvement, such as “helicopter parenting”, can undermine students’ autonomy and ability to manage stress, potentially hindering academic performance [52]. In the field of musicology, where doctoral students face the dual challenges of academic research and artistic practice, emotional support from parents is particularly crucial in fostering psychological resilience and emotional stability [41]. In the Chinese cultural context, parents’ strong emphasis on education often increases academic pressure on students [53], especially in fields like musicology, where career prospects are less certain. Therefore, exploring the impact of parental support on academic anxiety and academic performance holds considerable significance.

### 2.6. Time Management Skills

Time management refers to an individual’s ability to regulate themselves, allocate time effectively, and use it efficiently to achieve their goals. This concept is grounded in self-management theory, which emphasizes proactivity and self-discipline [54]. Students with strong time management skills are more successful in reducing academic anxiety and often achieve higher academic performance compared to those who struggle with time management [55]. In fact, time management has been shown to be a stronger predictor of college students’ GPA than standardized tests such as the SAT [56]. For doctoral students, time management is particularly critical, as they must not only navigate complex, long-term research projects but also balance multiple responsibilities, including teaching and research. Effective time management helps to alleviate academic stress and enhance scholarly productivity [57]. However, the relationship between time management and academic performance can vary based on cultural context and disciplinary differences [54]. In musicology, doctoral students face the dual challenge of managing heavy academic workloads alongside artistic practice, performance, and various academic activities, which heightens time pressure and psychological strain [58]. Therefore, examining the impact of time management skills on academic performance and academic anxiety among musicology doctoral students is of significant practical importance.

### 2.7. Facilitating Conditions

In this study, facilitating conditions refer to the resources, opportunities, and technical support that contribute to the academic development of doctoral students, encompassing access to academic resources and opportunities for training, technical equipment, and funding [59]. This concept originates from the Unified Theory of Acceptance and Use of Technology (UTAUT) and highlights the role of external environments in supporting individual behavior and achievement [60]. In the context of doctoral education, facilitating conditions provide students with critical resources, help alleviate academic pressure, and enhance research efficiency and overall well-being. Studies have shown that abundant academic resources (e.g., access to databases and libraries, along with advanced research equipment) can significantly improve doctoral students’ research productivity and output [61]. Moreover, interdisciplinary collaboration platforms foster innovation and elevate the quality of research [62], while sufficient research funding accelerates the progress of projects and betters the quality of their outcomes [63]. Additionally, improvements in facilitating conditions are closely tied to enhanced doctoral students’ well-being, because effective support systems help reduce academic anxiety [38]. In the field of musicology, doctoral students depend on academic resources and support for artistic practice (i.e., access to performance venues, artistic equipment, and creative opportunities), essential for balancing academic research with artistic expression [64]. Therefore, it is of importance to explore the impact of facilitating conditions on academic performance and doctoral students’ well-being.

### 2.8. Student Engagement

Student engagement refers to the time, effort, and resources that students invest to enhance their academic experience at a university [65]. Alexander Astin’s Involvement Theory argues that students learn and grow when they are actively involved in their academic and social environments, which is a key indicator of academic success and behavioral outcomes [66]. Student engagement can be divided into three dimensions: behavioral, emotional, and cognitive, which together contribute to academic achievement [67]. Research shows that the time and energy students dedicate to their academic pursuits are important predictors of academic performance and career development [68]. Moreover, student engagement is positively linked to overall well-being [69]. Conversely, low engagement is often associated with higher levels of academic anxiety, while appropriate levels of engagement, supported by external resources, can help reduce anxiety and boost academic confidence [70]. In doctoral education, sustained engagement and self-management skills are critical to success, with higher engagement levels typically correlating with better academic performance, mental health, and career preparedness [71]. In the field of musicology, behavioral engagement is reflected in participation in performances and research, cognitive engagement in a deep understanding of music theory, and emotional engagement is particularly significant due to its close connection with the emotional experience of music [72]. Thus, examining the influence of student engagement on academic performance, academic anxiety, and well-being is essential.

### 2.9. Academic Anxiety

Academic anxiety refers to the psychological pressure and tension students experience during academic tasks, often accompanied by physiological arousal and cognitive overload, which can negatively affect academic performance [73]. This psychology-based concept emphasizes the impacts of anxiety on emotions, cognition, and behavior [74]. In the education field, academic anxiety is seen in the forms of task-related worry, distraction, and reduced learning efficiency (e.g., during exams or complex research projects) [75]. Though moderate anxiety may serve as a learning motivator, excessive anxiety can impair memory, disrupt focus, and ultimately diminish academic performance [76]. Doctoral students face long-term academic pressure from independent research and high expectations and can be vulnerable to anxiety [77]. Academic anxiety is closely related to academic performance and well-being. Excessive anxiety potentially causes emotional exhaustion and psychological distress [50]. Strengthened self-efficacy and improved time management skills can help mitigate anxiety, and support from mentors can also effectively reduce anxiety levels [78]. In musicology, academic anxiety can be large since doctoral students need to master complex theories and performance techniques, and consequently, their academic burden is elevated [78]. In performance, anxiety can exacerbate self-doubt and undermine performance consistency and academic outcomes [79]. Therefore, it is critical to examine the influence of academic anxiety on the academic performance and well-being of musicology doctoral students in China.

### 2.10. Well-Being

Well-being comprises both emotional and cognitive dimensions. The emotional aspect involves balancing positive and negative emotions, while the cognitive aspect centers on an individual’s evaluation of life satisfaction [80]. According to Diener’s theory of subjective well-being, well-being is composed of positive emotions, life satisfaction, and a reduction in negative emotions [81]. It is closely linked to various life domains, such as interpersonal relationships, sense of purpose, and personal development [81]. Individuals with higher levels of well-being generally achieve better academic outcomes [82]. Specifically, positive emotions broaden cognitive perspectives, promote creativity, and thereby strengthen academic performance [83]. Additionally, emotional support from educators positively influences students’ mental health and academic engagement and improves their academic performance [84]. For doctoral students, well-being is important for managing academic stress, sustaining a positive outlook, and increasing productivity in independent research and long-term projects [85]. For musicology doctoral students, well-being is important in enhancing their expressiveness and creativity in performance and composition tasks and can lead to greater academic achievements [86]. Given the substantial peer-review pressure in musicology research, it is vital to maintain well-being for mental health and academic success [86]. In China, musicology doctoral students face additional challenges (e.g., intense academic competition, limited resources, and inadequate mentor support) that may further negatively impact their well-being [48]. Therefore, an exploration of well-being contributes to improved doctoral education and provides valuable insights into enhancing the quality of education in musicology.

## 3. Hypothesis Development

**Figure 1:**
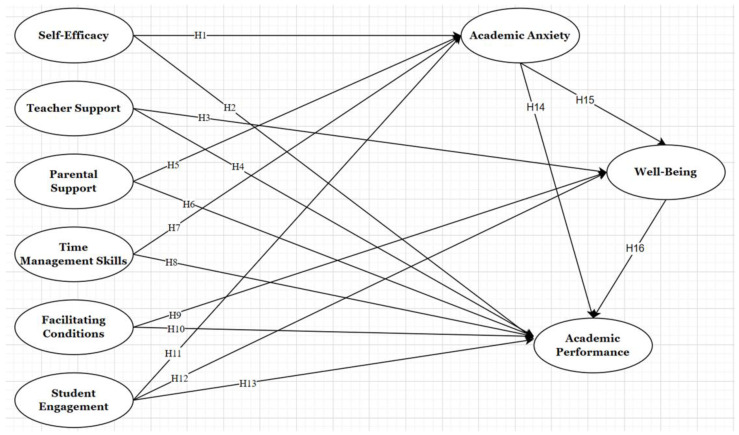
Developing a model for factors that affect doctoral students’ academic performance in musicology.

The literature review, as outlined in Table 1 and Figure 1, presents 16 initial hypotheses encompassing six independent variables, two mediating variables, and one dependent variable.

## 4. Methodology

### 4.1. Development of Instruments and Collection of Data

Our study seeks to understand and identify the key factors that impact the academic performance of music doctoral students, examining how these factors influence their academic results. Key focus areas include self-efficacy, teacher support and parental support, time management skills, facilitating conditions, student engagement, academic anxiety, and well-being. Based on a literature review, an empirical model was crafted, which guided the development of a questionnaire. For a robust data collection, 213 music doctoral students from various Chinese universities answered the questionnaire. If participants completed the questionnaire unusually quickly or did not thoroughly complete it, their data were excluded to maintain data integrity. This study adopted quantitative methods (i.e., Structural Equation Modeling (SEM)) to analyze the relationships between the variables. The goal was to determine how these factors affect musicology doctoral students’ academic performance, which provides insights that help shape educational policies and enhance student support services.

#### 4.1.1. Design of the Questionnaire

The instrument development and data collection process underwent delicate planning and multiple adjustments to guarantee data precision and study integrity. We constructed a questionnaire containing 37 questions based on an empirical model obtained from previous literature. This questionnaire used a 5-point Likert scale, and responses ranged from 1 (strongly disagree) to 5 (strongly agree). Each question was derived from prior studies and was modified to suit the specific needs of this research better to enhance its practicality. After the questionnaire was designed, five specialists in the field were invited to assess the content. Their insights led to modifications to ensure the questionnaire’s content validity and suitability for this study. Additionally, we carried out a preliminary pilot test before its formal deployment to confirm that the questionnaire genuinely reflected the participants’ real conditions. This pilot test facilitated the evaluations of the questions’ clarity and the questionnaire’s usability. We maintained strict process controls to ensure the data accuracy and the reliability of research findings.

The questionnaire development underwent delicate planning and multiple adjustments to maintain data accuracy and study integrity. The questions were customized to fit specific study goals and adapted from established scales related to the field to increase their relevance. Afterward, the experts in the field evaluated the questionnaire, leading to some modifications to improve content validity and relevance. A pilot test ensured the questionnaire accurately reflected the participants’ experiences, assessing item clarity and the overall operational effectiveness of the questionnaire. The survey reliability adopted Cronbach’s alpha, showing values greater than 0.7 for all scales, indicating a high internal consistency level. The structure of the questionnaire was also validated through factor analysis to ensure each indicator adequately represented the intended latent variables.

The questionnaire comprised two sections: the first collected basic demographic information from participants, including gender, age, academic year, and marital status. The second section measured nine research variables: self-efficacy (4 items), teacher support (5 items), parental support (3 items), time management skills (4 items), facilitating conditions (3 items), student engagement (4 items), academic anxiety (6 items), well-being (3 items), and academic performance (5 items). Designed to be concise and take approximately 13 min to complete, the questionnaire aims to minimize the burden on participants while gathering comprehensive data. Full details of the questionnaire and the specific items for each variable are provided in the Supplementary Materials.

#### 4.1.2. Process of Data Collection

In March 2024, this study employed a random sampling method, utilizing the Wenjuanxing platform to create an electronic questionnaire link, which was distributed via WeChat and email to ensure broad outreach to the target population. Specifically, school administrators and heads of teaching and research groups directed the questionnaire link to musicology doctoral students across various universities in China. To ensure the randomness of the sampling process, musicology departments or relevant units at each university followed a pre-established randomization procedure to distribute the questionnaire to their doctoral students, thereby ensuring a diverse and representative participant group. Participation was voluntary, with no mandatory screening criteria, thus minimizing selection bias and promoting a diverse and representative sample.

A total of 261 questionnaires were distributed, and 213 valid responses were collected, which gave a response rate of 81.6%. To improve the response rate, the research team sent reminder emails after the initial distribution to encourage participants who had not yet completed the questionnaire to respond. The sample size was strategically determined based on an efficacy analysis to achieve a statistical power of 0.80 at a medium effect size. This study did not employ any interim analyses or stopping rules, ensuring that the sample size collected was consistent with the initial plan. The participants’ demographic details are outlined in Table 2.

To safeguard participants’ privacy, all questionnaire responses were anonymized, and no personally identifiable information was requested. At the beginning of the survey, participants were provided with a detailed informed consent form outlining their rights, how their data would be used, and their ability to withdraw from this study at any time. The data collected were used exclusively for this study’s analysis and were securely stored on a password-protected server, accessible only to the research team. Furthermore, regular data backups were conducted to ensure the integrity and security of the data. Throughout the data collection and analysis process, we strictly adhered to the ethical guidelines approved by the Ethics Committee of South China Normal University, ensuring that participants’ rights and privacy were fully protected.

### 4.2. Data Processing and Analysis

The data collected for this study went through a thorough cleaning process to ensure accuracy. Initially, incomplete questionnaires were removed, and then we identified and eliminated responses with unusual answering patterns by calculating the standard deviation for each participant’s responses. Further, outliers significantly deviating from normal ranges were excluded using the interquartile range (IQR) method via boxplot analysis. After this process, 213 valid data sets were retained, ensuring the reliability of the subsequent analysis.

Descriptive statistical analysis was first performed using SPSS to present the sample demographic features and the distribution of key variables. Subsequently, Partial Least Squares Structural Equation Modeling (PLS-SEM) was conducted using SmartPLS 4.0 to assess how factors such as self-efficacy, teacher and parental support, time management skills, facilitating conditions, student engagement, academic anxiety, and well-being affect musicology doctoral students’ academic performance.

In this study, based on theoretical and methodological considerations, Partial Least Squares Structural Equation Modeling (PLS-SEM) was chosen as the data analysis method over other statistical approaches (e.g., Covariance-Based Structural Equation Modeling (CB-SEM) or traditional regression analysis). Firstly, this research examines relationships between variables (e.g., self-efficacy, parental support, time management, and student engagement), and their impact on the academic performance of musicology doctoral students. These relationships are previously underexplored, and the theoretical foundation is not fully established. PLS-SEM is particularly well suited to such exploratory studies since it allows for flexible modeling of complex relationships even when the theoretical framework is still developing [87]. Secondly, the primary objective of this study is to predict and explain the influence of multiple factors (e.g., self-efficacy and parental support) on academic performance. PLS-SEM is ideal for prediction-oriented research, as it maximizes the explained variance of the dependent variables and effectively explains and predicts academic performance and mental health [88]. Additionally, PLS-SEM performs well with smaller sample sizes, and given this study’s relatively limited sample size, it is a more appropriate analytical tool [89]. Furthermore, PLS-SEM is capable of handling non-normally distributed data, given the study population (Chinese doctoral students in musicology) and the sensitive issues examined (such as academic anxiety) where data distributions may be skewed. PLS-SEM’s ability to manage non-normal data is therefore crucial [90]. Finally, PLS-SEM can simultaneously estimate complex relationships between multiple independent and dependent variables and supports both formative and reflective constructs, which is essential for capturing the intricate interactions among the various factors in this study [91]. Moreover, PLS-SEM is more robust than traditional regression methods when addressing multicollinearity, ensuring the accuracy of the analysis results [91]. Given these considerations, PLS-SEM aligns well with the exploratory nature, predictive aims, small sample size, and data characteristics of this study, making it the most appropriate analytical method.

This study used PLS-SEM to rigorously evaluate both the measurement and structural models. Composite Reliability (CR) and Cronbach’s alpha were used to assess the internal consistency of the measurement model. A CR value larger than 0.7 is considered strong internal consistency, a standard frequently adopted in empirical research to ensure the reliability of measurement scales [92]. The Average Variance Extracted (AVE) and factor loadings were examined for convergent validity. An AVE value greater than 0.5 suggests that the latent variable explains the indicator’s variance. Moreover, discriminant validity was assessed by comparison between the square root of the AVE and the constructs’ correlations. According to the Fornell–Larcker criterion, good discriminant validity can be confirmed by a square root of the AVE larger than the correlations between latent variables [93]. This study set the Heterotrait–Monotrait Ratio (HTMT) as a test, and an HTMT value below 0.90 indicates adequate discriminant validity between the latent variables [94]. HTMT assesses the distinctiveness between constructs by analyzing the ratio of the average heterotrait (between-construct) correlations to the average monotrait (within-construct) correlations [95]. Essentially, this value indicates the extent to which elements of the same construct are more closely related to each other than to elements of different constructs. In contrast, the Fornell–Larcker criterion gauges the uniqueness and discriminant validity of constructs by comparing the square root of each construct’s Average Variance Extracted (AVE) to its correlations with other constructs [96].

For the structural model evaluation, we mainly analyzed the path coefficients and the determination coefficient (R^2^). The path coefficients reflect the strength and direction of relationships between latent variables. *p*-values were used to verify the significance of these coefficients, with a value below 0.05 indicating statistical significance [97]. The R^2^ value measures how much the exogenous variables explain variance in the endogenous variables. Specifically, values near 0.25, 0.50, or 0.75 represent weak, moderate, or strong explanatory power, respectively. It is essential to interpret these R^2^ values within the context of the specific field of study and consider the complexity of the model. This study also calculated the Variance Inflation Factor (VIF) for all latent variables to check for multicollinearity, where a VIF of less than 5 is preferable, indicating no significant multicollinearity issues [98]. These steps collectively helped to systematically validate the research hypotheses and deepen the understanding of how various factors influence the students’ academic performance.

## 5. Results

Based on the descriptive statistics and measurement model outcomes, this research identified that teacher support (β = 0.206, *p* = 0.003), student engagement (β = 0.183, *p* = 0.007), academic anxiety (β = −0.206, *p* = 0.002), and well-being (β = 0.163, *p* = 0.003) each have direct influences on academic performance. Specifically, teacher support, student engagement, and well-being positively affect academic performance, whereas academic anxiety has a notable negative impact. Additional analyses showed that self-efficacy (β = −0.265, *p* = 0.000), parental support (β = −0.278, *p* = 0.000), time management skills (β = −0.174, *p* = 0.012), and student engagement (β = −0.161, *p* = 0.015) significantly reduce academic anxiety. Furthermore, teacher support (β = 0.195, *p* = 0.013), facilitating conditions (β = 0.278, *p* = 0.001), and student engagement (β = 0.191, *p* = 0.02) significantly boost the well-being of doctoral students. Academic anxiety adversely affects well-being (β = −0.173, *p* = 0.013), indicating a significant inverse correlation between them. This study’s structural model analysis and initial hypothesis testing pinpointed the key factors significantly impacting musicology doctoral students’ academic performance.

Descriptive statistics were employed to illustrate the characteristics of the respondents’ answers. According to prior research, the acceptable thresholds for skewness and kurtosis are |2.3| [99]. Thus, based on the data presented in Table 3, both skewness and kurtosis fall within these acceptable limits.

### Measurement Model Analysis

An extensive analysis of external loadings, internal consistency reliability, and convergent validity of variables showed that aside from a few indicators like AA4 with a lower loading of 0.688, all indicator loadings surpassed the commonly accepted standard of 0.7, affirming the effectiveness of the measurement items. The Average Variance Extracted (AVE) was larger than 0.50 for all variables, with the lowest at 0.611, indicating that each variable effectively explains more than half of the variance for its associated measurement indicators, in line with recommendations by Sarabia-Andreu et al. [100]. Additionally, the measurement model’s reliability and consistency were robustly confirmed as the Composite Reliability and Cronbach’s alpha for all examined variables exceeded the established minimum threshold of 0.70. The credibility of the measurement model is strengthened. Comprehensive details regarding the external loadings, reliability, and convergent validity of each variable are systematically compiled in Table 4. The compilation lays a strong empirical foundation for the validity and reliability of the measurement model adopted in this study.

This study utilized the Fornell–Larcker criterion (see Table 5) and the Heterotrait–Monotrait Ratio (HTMT) to evaluate the model’s discriminant validity [101]. Moreover, the largest value of HTMT is 0.896 (see Table 6), staying below the critical threshold of 0.90, thus further affirming the effective distinction between this study’s variables [102].

After the validity of the measurement model was confirmed, the next phase involved a detailed examination of the structural model. This included checking for collinearity, evaluating R^2^ values, and assessing the path coefficients’ significance. A significance level of 0.05 was utilized, as well as a bias-corrected non-parametric bootstrap approach [103].

As shown in Table 7, the collinearity test results revealed that Variance Inflation Factor (VIF) values were well below the acceptable threshold of 5 for all constructs. This confirms that multicollinearity was not an issue in the model [104]. This suggests minimal overlap between constructs and ensures multicollinearity does not distort the accuracy of the model. This supports the reliability of the variable estimates. Moreover, the discriminant validity of the model is solid, with VIF values showing the independence of the constructs, enhancing the robustness and applicability of the structural model.

**Figure 2:**
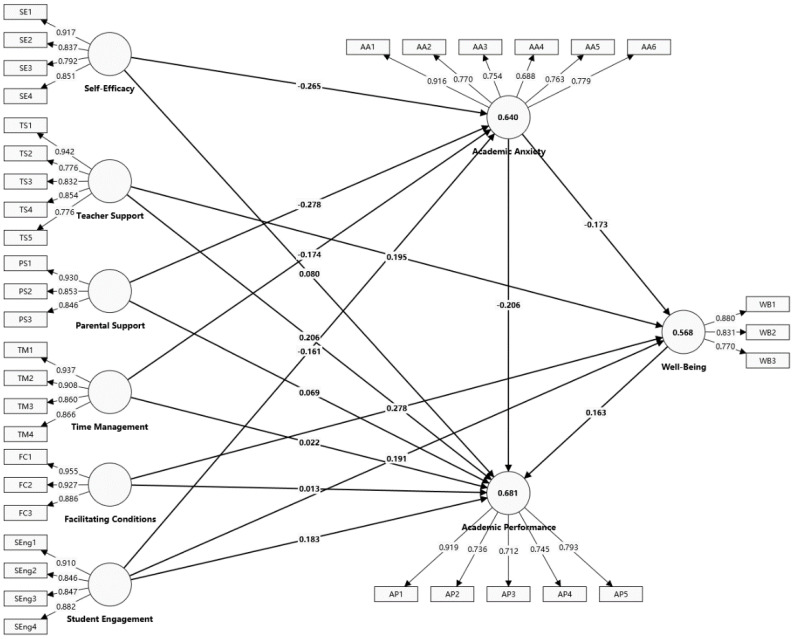
The evaluation results of the structural model with R^2^, path coefficients, and *p*-values.

Figure 2 indicates R^2^ values of 0.681 for academic performance, 0.640 for academic anxiety, and 0.568 for well-being. These R^2^ values reflect the proportion of variance explained by the model for each variable and highlight its effectiveness in clarifying these relationships. Specifically, the R^2^ value of 0.681 indicates the model explains over 68% of the variance in academic performance, while it explains 64% of the variance in academic anxiety and 56.8% in well-being. These outcomes validate the model’s strength in accurately describing the academic performance, academic anxiety, and well-being of doctoral students in musicology. Notably, all R^2^ values above 0.1 further reinforce the model’s statistical relevance and ability to predict key outcomes. Thus, the model is demonstrated to have robust explanatory and predictive power, efficiently identifying the principal elements that affect musicology doctoral students’ academic results.

According to the data in Table 8, out of 16 hypotheses tested, 12 received empirical support. This study compared the effects of various factors on well-being, academic anxiety, and academic performance. It was found that facilitating conditions had the most substantial positive influence on well-being (β = 0.278, *p* = 0.001), a stronger impact than teacher support (β = 0.195, *p* = 0.013). In reducing academic anxiety, parental support had the most pronounced negative effect (β = −0.278, *p* < 0.001), significantly outperforming the impacts of self-efficacy (β = −0.265, *p* < 0.001) and time management skills (β = −0.174, *p* = 0.012). Concerning academic performance, both the negative influence of academic anxiety (β = −0.206, *p* = 0.002) and the positive effect of teacher support (β = 0.206, *p* = 0.003) were identified as key factors, highlighting the equal importance of reducing academic anxiety and enhancing educator support in improving academic performance. In sum, this research underscores the critical roles that facilitating conditions and parental support play in boosting well-being and reducing academic anxiety, while the alleviation of academic anxiety and reinforcement of teacher support are essential in advancing musicology doctoral students’ academic performance. Moreover, Figure 2 presents the final model, including R^2^ values, path coefficients, and their corresponding *p*-values.

Based on the results from the indirect effects analysis (as shown in Table 9), parental support, self-efficacy, and facilitating conditions play significant mediating roles in influencing students’ academic performance and mental health. Parental support, by lessening academic anxiety, not only significantly boosts students’ academic performance (β = 0.057, *p* = 0.007) but also considerably enhances their well-being (β = 0.048, *p* = 0.024), indicating the critical role parents play in both academic and psychological aspects of student life. Similarly, self-efficacy, by reducing academic anxiety, significantly improves academic performance (β = 0.055, *p* = 0.010) and psychological well-being (β = 0.046, *p* = 0.031), underscoring its essential role in alleviating academic burdens and promoting mental health. Additionally, facilitating conditions, by enhancing students’ well-being (β = 0.045, *p* = 0.028), subsequently significantly improve academic performance, suggesting that a supportive academic environment and resources can foster academic success by enhancing well-being.

## 6. Discussion

This study delves into the critical factors that shape the academic performance of doctoral students in musicology, drawing from social cognitive theory. This research focuses on variables such as self-efficacy, teacher support, parental support, time management skills, enabling conditions, student engagement, academic anxiety, and well-being. While these factors are well explored across various academic fields, there is a noticeable paucity of such focused studies within musicology doctoral programs. To bridge this gap, this study utilized Structural Equation Modeling (PLS-SEM) to analyze data collected from 213 musicology doctoral candidates, thereby testing the proposed theoretical hypotheses. Out of 16 hypotheses, 12 were substantiated. While the model is grounded in established literature, the findings paralleled some previous studies but diverged from others in notable ways.

The analysis confirmed that self-efficacy significantly reduces academic anxiety, aligning with the foundational theory of self-efficacy posited by Bandura and Locke [105], which suggests that boosting an individual’s ability to manage stress can effectively decrease anxiety. However, unlike previous findings, this study did not find a significant positive impact of self-efficacy on academic performance [106], a deviation that suggests that while cognitive and learning competencies are crucial, they might be less effective when isolated [107]. Highly efficacious students often see challenges as growth opportunities and are strategic in problem-solving and managing stress [108]. This study speculates that for musicology doctoral students, the skills in creativity and performance might be more influential in determining academic success, which could overshadow the direct impact of self-efficacy. Nevertheless, self-efficacy indirectly supported academic performance by reducing academic anxiety. According to research by Putwain [109], lowering academic anxiety can improve learning efficiency and academic performance as anxiety consumes working memory, particularly in complex cognitive tasks, thus adversely affecting academic performance [110]. Furthermore, this study revealed that self-efficacy enhanced doctoral students’ well-being by lessening academic anxiety, corroborating findings by Cao et al. [111]. Considering the rising trend of delayed graduations among doctoral students in China—from 39.7% in 2017 to 49.4% in 2021—the intense pressures of doctoral education today might necessitate a reevaluation of overly stringent academic performance standards to reduce academic anxiety. Therefore, it is recommended that educational leaders focus on enhancing doctoral students’ self-efficacy to decrease their academic anxiety, thereby indirectly improving their academic performance and psychological health. This approach is crucial for student development and aligns with the broader public health goal of reducing psychological distress.

The findings of this study illuminate the beneficial effects of teacher support on the subjective well-being and scholarly success of music doctoral students. Initially, the emotional support and academic mentorship provided by faculty significantly boost doctoral students’ well-being, in alignment with the fundamental concepts of Self-Determination Theory. Henning and colleagues [112] argue that an individual’s well-being is contingent upon satisfying their needs for autonomy, competence, and relatedness. Further studies highlight that in environments characterized by academic pressure, the support and understanding from teachers play an essential role, markedly improving students’ well-being [113]. By nurturing a sense of belonging and security, teacher support not only aids doctoral students in managing stress but also positively affects their overall graduate experience [114]. Additionally, the role of teacher support extends to academic performance. The instructional feedback and mentorship from educators enhance the academic capabilities and development of doctoral students [115]. Research has shown that emotional support can significantly increase doctoral students’ academic engagement and performance by enhancing their motivation to learn and sense of self-efficacy [116]. A positive teacher–student relationship has also been identified as a predictor of doctoral students’ academic achievements [117]. These results emphasize the critical role of constructive teacher–student interactions in fostering academic growth and suggest that universities should strengthen doctoral students’ psychological well-being and academic skills through customized support and feedback mechanisms, thus promoting overall public health.

Significantly, parental support inversely correlates with academic anxiety among doctoral students in musicology, indicating that stronger parental support is linked to reduced academic anxiety. This aligns with findings by Cutrín et al. [118], who noted that democratic parenting significantly lowers anxiety levels in students. Yet, other studies suggest that certain parental behaviors, such as excessive control, might exacerbate anxiety disorders, indicating that the effects of different types of parental support on alleviating academic anxiety can vary. While parental support is shown to reduce academic anxiety effectively, this study did not observe a direct positive influence on academic performance, contrasting with findings by Rodriguez-Oramas et al. [119], who found that a family environment emphasizing learning opportunities dramatically enhances students’ intrinsic academic motivation. Additionally, social support is known to boost self-esteem and decrease depression effectively [120]. The findings of this study are potentially reflective of the intense academic environment in China, where doctoral students’ performance tends to depend more on individual efforts, advisor support, and research resources, and the direct impact of parental emotional support on academic performance is relatively minor [121]. This research also identified academic anxiety as a mediator between parental support and doctoral students’ well-being and academic performance, supporting the theoretical model by Zeng et al. [122], which posits that social support can enhance well-being by mitigating stress and anxiety. Moreover, this aligns with research by Sun et al. [123], which underscores the pivotal role of emotional support in stress reduction and mental health enhancement. Although this study focused on musicology doctoral students, its conclusions may be relevant to other disciplines within higher education. Universities could benefit from strengthening collaboration between homes and academic institutions and expanding mental health services to assist students in managing academic anxiety. This approach would foster academic achievement and enhance students’ psychological well-being, contributing to broader societal gains.

This research confirms that effective time management is inversely related to academic anxiety among musicology doctoral students, consistent with previous studies. Tsitsia et al. [124] emphasize that strong time management skills help students allocate and use their time efficiently, reducing the stress caused by a buildup of tasks. Sverdlik [30] also highlights the importance of prioritizing key academic tasks as a critical time management strategy that helps minimize anxiety from handling multiple responsibilities. While existing literature suggests that effective time management is vital for reducing academic anxiety in students facing numerous deadlines and high expectations, this study did not find direct evidence supporting a link between time management and improved academic performance, as proposed by Ariastuti and Wahyudin [125]. This suggests that other factors, such as creativity and cognitive load, may influence how time management affects academic performance in musicology doctoral students. Mediation analysis showed that lowering academic anxiety could indirectly enhance academic performance, supporting findings from Pravita and Kuswandono [126], who reported a negative association between time management skills and state–trait anxiety, a key factor that negatively impacts psychological health and academic success. These insights are applicable across various doctoral student groups, particularly in fields that require extensive creative thinking and independent research. Education leaders are encouraged to provide time management training to doctoral students to better manage multitasking demands, thereby reducing academic anxiety and enhancing academic success rates. Such initiatives could potentially reduce the negative impact of academic anxiety on students’ mental health, improving the overall public health profile among the doctoral student population.

Furthermore, this study reaffirms that favorable facilitating conditions boost the well-being of musicology doctoral students, supporting existing literature [127] on the positive role of external resources in enhancing mental health and well-being. Grounded in Self-Determination Theory, external resources are shown to elevate life satisfaction by meeting essential psychological needs for autonomy, competence, and belonging. Yet, contrary to the implications from Realyvásquez-Vargas et al. [128] that facilitating conditions can indirectly enhance academic performance by improving the educational environment, this study did not observe a direct impact on academic performance. This suggests that doctoral students in musicology may view academic performance as more dependent on intrinsic factors such as academic interest, motivation, and quality of academic supervision rather than merely on external resources. Additionally, this study highlights the role of well-being as a mediator between facilitating conditions and academic performance, resonating with the views of Ngui and Lay [129] that improved conditions foster academic resilience and stress management capabilities, thus promoting academic success. Well-being, as a vital psychological asset, aids doctoral students in managing academic challenges and maintaining high levels of engagement and motivation under stress [130]. Although facilitating conditions did not directly influence academic performance, they provide a psychological base for academic success by enhancing well-being. Hence, universities should prioritize infrastructure enhancement, emotional support, and academic mentoring to boost doctoral students’ well-being and academic achievements.

This research confirms that engagement among musicology doctoral students is crucial for academic performance, reinforcing existing scholarly findings. Enhanced student engagement is notably linked to decreased levels of academic anxiety, supporting Adams et al.’s assertion [131] that highly engaged students generally enjoy better mental health and experience lower anxiety. Furthermore, this study finds that student engagement benefits well-being, aligning with Hidayat et al. [132], who argue that involvement in activities with shared goals boosts positive emotions, thereby deepening learning engagement and elevating both subjective and overall happiness. Additionally, a robust connection exists between student engagement and improved academic performance, corroborating Knifsend’s findings [133] that engaged students perform better academically and enjoy higher levels of well-being. Kim [134] highlights that the dedication of time and energy to meaningful educational activities is a key indicator of students’ academic and professional progress. Moreover, this study identifies well-being as an important mediator between student engagement and academic performance, supporting the research by Cabellos et al. [59], which shows that higher well-being can protect against burnout and sustain long-term academic performance. Positive emotions such as well-being enhance cognitive processes like creativity and problem-solving, vital for academic success [135]. Consequently, universities should provide more interactive learning opportunities to elevate doctoral students’ engagement and well-being, thereby fostering academic success and mental health. This is crucial for enhancing the mental well-being of doctoral students and the overall academic milieu, aligning with public health goals to improve general well-being.

Lastly, this study unequivocally shows that high levels of academic anxiety adversely impact doctoral students’ academic performance, echoing findings from the existing literature. Research indicates that academic anxiety can undermine cognitive functions, reduce motivation for learning, and ultimately affect academic performance [136]. Specifically, students with high anxiety levels often have difficulty concentrating and are prone to self-doubt, which negatively affects their academic task performance [137]. Additionally, this research discovers that academic anxiety impacts academic results and significantly correlates negatively with well-being. As academic pressures and anxiety rise, students are more likely to experience negative emotions, reducing their overall sense of well-being. This supports previous research on the harmful effects of anxiety on mental health [138]. Importantly, students with higher levels of well-being demonstrate superior academic performance, consistent with past studies showing that well-being is linked to more positive attitudes towards learning, higher motivation, and better coping skills for academic challenges [139]. Well-being provides emotional support to students and helps them focus on academic tasks, resulting in better academic achievements. Furthermore, this study shows that well-being acts as a psychological buffer, mediating the effects of anxiety on academic performance, validating its role in offsetting the negative impacts of academic anxiety [140]. These insights suggest that educators should focus on enhancing doctoral students’ well-being through mental health support to mitigate the adverse effects of academic anxiety on performance.

## 7. Contribution and Implications

This study deepens the understanding of factors leading to musicology doctoral students’ academic success in China. It explores how individual, social, and environmental factors interactively affect students’ academic performance. This study broadens the scope of doctoral education research, previously dominated by natural sciences and engineering studies, and extends it into the humanities and arts. A pivotal aspect of this research is its exploration of the profound impact psychological factors have on doctoral students’ academic performance (i.e., self-efficacy, academic anxiety, well-being, and their complex interrelations).

Using Partial Least Squares Structural Equation Modeling (PLS-SEM), this research highlights that academic anxiety worsens academic performance. At the same time, factors have significant positive effects (i.e., teacher support, student engagement, and well-being). These results agree with social cognitive theory and Self-Determination Theory. The essential roles of external support and internal motivation in fostering doctoral student success are emphasized in the fields of creativity and performance. The link is more pronounced between mental health and academic success. This study shows factors (i.e., self-efficacy, parental support, time management skills, and student engagement) crucial in lowering academic anxiety and indirectly enhancing academic performance. Furthermore, the positive roles of teacher support, conducive learning environments, and active student engagement in boosting doctoral students’ well-being are found. The correlation between well-being and academic anxiety was negative. Our study sheds new light on the interplay between a doctoral student’s psychological state and academic achievements.

Practically, the findings suggest that higher education institutions should focus on increasing doctoral students’ self-efficacy, provide training in time management, and enhance mental health support to reduce academic anxiety and improve both academic performance and well-being. Given the growing global demand for doctoral education, these measures are recommended to help optimize the educational settings, support structures, and mental health provisions for doctoral students. These insights are relevant to China and other developing areas and offer valuable guidance for the global higher education community, particularly within doctoral programs in the arts and creative sectors. This study lays a solid empirical foundation for educational policymakers to improve the quality of doctoral education.

## 8. Limitations and Future Directions

This study offers valuable insights but also has certain limitations. First, the data are drawn exclusively from musicology doctoral students in China, which may limit the generalizability of the findings to other cultural and academic contexts. Different cultures have distinct views on mental health, social support, and academic pressure, as well as different coping strategies. Therefore, future research should include more diverse samples and conduct cross-cultural comparisons to validate the broader applicability of these findings. Second, the reliance on cross-sectional data restricts the ability to establish causal relationships. Longitudinal studies would provide a deeper understanding of the dynamic interactions between psychological factors, support systems, and academic performance, particularly how these factors evolve over the course of doctoral study. Additionally, self-reported data may introduce social desirability bias. To improve the objectivity and accuracy of the data, future research could integrate both qualitative and quantitative methods, such as in-depth interviews or observational studies. Furthermore, this study did not consider factors such as financial pressure, research resources, and policy support, which could significantly impact doctoral students’ academic success. In the future, these variables should be incorporated for developing a more comprehensive student support framework. Lastly, the theoretical framework used in this study could be applied to other disciplines to investigate the effects of factors such as teacher support and self-efficacy among doctoral students in different academic fields so that more targeted support strategies can be offered.

In conclusion, future research should improve sample diversity, validate causal relationships, and diversify data collection methods to enhance the generalizability and rigor of the findings. These improvements would provide a stronger theoretical foundation and practical guidance for advancing educational policies and student support systems.

## 9. Conclusions

This study employed Partial Least Squares Structural Equation Modeling (PLS-SEM) to investigate the impact of multiple factors on the academic performance of musicology doctoral students in China. The findings highlight the positive influence of factors (e.g., teacher support, student engagement, and well-being) on academic performance and the significant negative effect of academic anxiety. Notably, teacher support enhances academic outcomes by boosting students’ well-being and is key in mitigating academic anxiety. This suggests that teachers’ guidance and emotional support have dual functions of fostering psychological well-being and academic success. Additionally, this study revealed that self-efficacy, parental support, time management skills, and student engagement effectively reduce academic anxiety and underscored the importance of personal behaviors and social support in managing doctoral students’ mental health. This research identified a significant negative relationship between academic anxiety and well-being and emphasized the importance of reducing academic pressure for improving students’ overall mental health. Well-being further acts as a mediator between access to resources and academic performance. Thus, it shows that external resources can enhance students’ well-being and improve academic performance by bettering psychological health. These findings highlight the need to provide doctoral students with comprehensive psychological support and readily accessible resources.

This study offers valuable empirical evidence for educational leaders and policymakers. Educational institutions can enhance teacher guidance, foster parental emotional support, and offer time management training to effectively reduce students’ academic anxiety, elevate well-being, and improve academic performance. Furthermore, improving access to academic resources and creating favorable learning environments positively impact students’ well-being and have implications for their long-term development. This study provides important insights into the academic success and mental health of doctoral students, but there are limitations. The sample size and specific cultural context may limit the generalizability of the findings. Future research should include cross-disciplinary and cross-cultural validation, as well as longitudinal studies to examine the long-term effects of these factors so that the robustness and applicability of the results can be enhanced. Overall, this study not only contributes new empirical evidence to the field of musicology doctoral student research but also provides strong support for shaping global higher education policies aimed at improving the psychological well-being and academic success of doctoral students.

## Figures and Tables

**Table 1:** List of hypotheses.

Hypotheses	Description
H1	Self-efficacy negatively influences musicology doctoral students’ academic anxiety.
H2	Self-efficacy positively influences musicology doctoral students’ academic performance.
H3	Teacher support positively influences musicology doctoral students’ well-being.
H4	Teacher support positively influences musicology doctoral students’ academic performance.
H5	Parental support negatively influences musicology doctoral students’ academic anxiety.
H6	Parental support positively influences musicology doctoral students’ academic performance.
H7	Time management skills negatively influence musicology doctoral students’ academic anxiety.
H8	Time management skills positively influence musicology doctoral students’ academic performance.
H9	Facilitating conditions positively influence musicology doctoral students’ well-being.
H10	Facilitating conditions positively influence musicology doctoral students’ academic performance.
H11	Student engagement negatively influences musicology doctoral students’ academic anxiety.
H12	Student engagement positively influences musicology doctoral students’ well-being.
H13	Student engagement positively influences musicology doctoral students’ academic performance.
H14	Academic anxiety negatively influences musicology doctoral students’ academic performance.
H15	Academic anxiety negatively influences musicology doctoral students’ well-being.
H16	Well-being positively influences musicology doctoral students’ academic performance.

**Table 2:** Participants’ demographic information.

Demographic Basics		Number	Percent
Age	20–24 years old	52	24.40%
	25–29 years old	127	59.60%
	30 years old and above	34	16%
Gender	Female	129	60.60%
	Male	84	39.40%
Marital status	Married	137	64.30%
	Not yet married	76	35.70%
Job status	Not yet working	168	78.90%
	Already working	45	21.10%
Academic year	First year	17	8.00%
	Second year	82	38.50%
	Third year	68	31.90%
	Fourth year and above	46	21.60%

**Table 3:** Constructs, indicators, skewness, and kurtosis examined in this study.

Constructs	Indicator	Skewness	Kurtosis
		Statistic	Statistic
Self-Efficacy	SE1: Satisfying requirements	−0.213	−0.212
	SE2: Excellent outcomes	−0.701	0.475
	SE3: Skilled presentations	−0.506	0.239
	SE4: Preparedness	−0.518	0.030
Teacher Support	TS1: Clear standards	−0.205	−0.373
	TS2: Prompt response	−0.326	0.214
	TS3: Equal treating	−0.542	0.252
	TS4: Supporting decisions	−0.210	−0.132
	TS5: Promoting collaborations	−0.256	−0.150
Parental Support	PS1: Financial support	−0.206	−0.420
	PS2: Understanding and encouraging	−0.336	0.025
	PS3: Emotional support	−0.657	0.433
Time Management Skills	TM1: Prioritizing tasks	−0.075	−0.548
	TM2: Strict schedules	−0.622	0.067
	TM3: Downtime management	−0.546	0.122
	TM4: Effective multitasking	−0.520	0.220
Facilitating Conditions	FC1: Helpful people	−0.152	−0.548
	FC2: Access to resources	−0.475	−0.185
	FC3: Workshops and training sessions	−0.617	−0.021
Student Engagement	SEng1: Intense focus	−0.078	−0.356
	SEng2: Making best effort	−0.566	0.699
	SEng3: No withdrawal intention	−0.832	0.937
	SEng4: Great satisfaction	−0.472	0.360
Academic Anxiety	AA1: Experiencing anxiety	−0.071	−0.510
	AA2: Worries about progress	−0.345	0.352
	AA3: Excessive worries about outcomes	0.006	0.063
	AA4: Difficult to relax	−0.265	0.434
	AA5: Significant unease	−0.341	0.364
	AA6: Feeling irritable or frustrated	−0.342	0.659
Well-Being	WB1: Pleased with progress	−0.335	−0.335
	WB2: Growing in research and challenges	−0.300	−0.415
	WB3: Meeting expectations	−0.163	−0.562
Academic Performance	AP1: Improving academic skills	−0.216	−0.593
	AP2: Advancing writing skills	−0.044	−0.558
	AP3: Confident showcasing	0.049	−0.764
	AP4: Advancing critical thinking abilities	0.052	−0.749
	AP5: Acquiring knowledge	0.023	−0.818

**Table 4:** Constructs, indicators, outer loadings, convergent validity, and reliability examined in this study.

Constructs	Indicator	Outer Loadings	Cronbach’s Alpha	Average Variance Extracted (AVE)	Composite Reliability
Self-Efficacy	SE1: Satisfying requirements	0.917	0.872	0.723	0.912
	SE2: Excellent outcomes	0.837			
	SE3: Skilled presentations	0.792			
	SE4: Preparedness	0.851			
Teacher Support	TS1: Clear standards	0.942	0.893	0.703	0.922
	TS2: Prompt response	0.776			
	TS3: Equal treating	0.832			
	TS4: Supporting decisions	0.854			
	TS5: Promoting collaborations	0.776			
Parental Support	PS1: Financial support	0.93	0.85	0.769	0.909
	PS2: Understanding and encouraging	0.853			
	PS3: Emotional support	0.846			
Time Management Skills	TM1: Prioritizing tasks	0.937	0.915	0.798	0.94
	TM2: Strict schedules	0.908			
	TM3: Downtime management	0.86			
	TM4: Effective multitasking	0.866			
Facilitating Conditions	FC1: Helpful people	0.955	0.913	0.852	0.945
	FC2: Access to resources	0.927			
	FC3: Workshops and training sessions	0.886			
Student Engagement	SEng1: Intense focus	0.91	0.894	0.76	0.927
	SEng2: Making best effort	0.846			
	SEng3: No withdrawal intention	0.847			
	SEng4: Great satisfaction	0.882			
Academic Anxiety	AA1: Experiencing anxiety	0.916	0.871	0.611	0.903
	AA2: Worries about progress	0.77			
	AA3: Excessive worries about outcomes	0.754			
	AA4: Difficult to relax	0.688			
	AA5: Significant unease	0.763			
	AA6: Feeling irritable or frustrated	0.779			
Well-Being	WB1: Pleased with progress	0.88	0.772	0.686	0.867
	WB2: Growing in research and challenges	0.831			
	WB3: Meeting expectations	0.77			
Academic Performance	AP1: Improving academic skills	0.919	0.841	0.615	0.888
	AP2: Advancing writing skills	0.736			
	AP3: Confident showcasing	0.712			
	AP4: Advancing critical thinking abilities	0.745			
	AP5: Acquiring knowledge	0.793			

**Table 5:** Results from the Fornell–Larcker test for evaluating discriminant validity.

	Academic Anxiety	Academic Performance	Facilitating Conditions	Parental Support	Self-Efficacy	Student Engagement	Time Management Skills	Teacher Support	Well-Being
Academic Anxiety	0.781								
Academic Performance	−0.725	0.785							
Facilitating Conditions	−0.750	0.707	0.923						
Parental Support	−0.745	0.729	0.764	0.877					
Self-Efficacy	−0.730	0.703	0.791	0.758	0.850				
Student Engagement	−0.705	0.738	0.762	0.787	0.734	0.872			
Time Manage-ment Skills	−0.724	0.708	0.788	0.799	0.782	0.750	0.893		
Teacher Support	−0.718	0.744	0.743	0.778	0.713	0.780	0.756	0.838	
Well-Being	−0.656	0.689	0.698	0.701	0.685	0.677	0.712	0.675	0.828

**Table 6:** Evaluation of discriminant validity obtained from the HTMT test.

	Academic Anxiety	Academic Performance	Facilitating Conditions	Parental Support	Self-Efficacy	Student Engagement	Time Management Skills	Teacher Support	Well-Being
Academic Anxiety									
Academic Performance	0.826								
Facilitating Conditions	0.824	0.789							
Parental Support	0.840	0.846	0.856						
Self-Efficacy	0.812	0.796	0.876	0.863					
Student Engagement	0.778	0.836	0.834	0.890	0.817				
Time Management Skills	0.794	0.792	0.853	0.896	0.866	0.820			
Teacher Support	0.799	0.842	0.811	0.878	0.790	0.860	0.828		
Well-Being	0.772	0.830	0.816	0.859	0.820	0.803	0.837	0.793	

**Table 7:** Variance inflation factor values of various constructs.

	Academic Anxiety	Academic Performance	Facilitating Conditions	Parental Support	Self-Efficacy	Student Engagement	Time Management Skills	Teacher Support	Well-Being
Academic Anxiety		3.001							2.704
Academic Performance									
Facilitating Conditions		4.026							3.211
Parental Support	3.754	4.206							
Self-Efficacy	3.108	3.622							
Student Engagement	3.109	3.655							3.239
Time Management Skills	3.612	4.089							
Teacher Support		3.518							3.158
Well-Being		2.487							

**Table 8:** Results of the initial hypothesis test.

Relationships	Path Coefficients (β)	Sample Mean	Standard Deviation	*p*-Values	T-Statistics	Result
H1: Self-Efficacy -> Academic Anxiety	−0.265	−0.268	0.068	0	3.88	Supported
H2: Self-Efficacy -> Academic Performance	0.08	0.082	0.076	0.147	1.051	Not Supported
H3: Teacher Support -> Well-Being	0.195	0.197	0.087	0.013	2.241	Supported
H4: Teacher Support -> Academic Performance	0.206	0.206	0.075	0.003	2.759	Supported
H5: Parental Support -> Academic Anxiety	−0.278	−0.275	0.07	0	3.97	Supported
H6: Parental Support -> Academic Performance	0.069	0.074	0.091	0.225	0.756	Not Supported
H7: Time Management Skills -> Academic Anxiety	−0.174	−0.175	0.077	0.012	2.248	Supported
H8: Time Management Skills -> Academic Performance	0.022	0.019	0.08	0.393	0.272	Not Supported
H9: Facilitating Conditions -> Well-Being	0.278	0.278	0.093	0.001	3.002	Supported
H10: Facilitating Conditions -> Academic Performance	0.013	0.008	0.084	0.438	0.157	Not Supported
H11: Student Engagement -> Academic Anxiety	−0.161	−0.161	0.074	0.015	2.168	Supported
H12: Student Engagement -> Well-Being	0.191	0.187	0.093	0.02	2.061	Supported
H13: Student Engagement -> Academic Performance	0.183	0.183	0.074	0.007	2.454	Supported
H14: Academic Anxiety -> Academic Performance	−0.206	−0.206	0.072	0.002	2.887	Supported
H15: Academic Anxiety -> Well-Being	−0.173	−0.178	0.078	0.013	2.222	Supported
H16: Well-Being -> Academic Performance	0.163	0.165	0.058	0.003	2.801	Supported

**Table 9:** Results of indirect effect analysis.

Relationships	Path Coefficients (β)	Sample Mean	Standard Deviation	*p*-Values	T-Statistics	Result
Teacher Support -> Well-Being -> Academic Performance	0.032	0.033	0.020	0.055	1.600	Not Supported
Time Management Skills -> Academic Anxiety -> Well-Being -> Academic Performance	0.005	0.005	0.004	0.091	1.337	Not Supported
Time Management Skills -> Academic Anxiety -> Well-Being	0.030	0.031	0.021	0.072	1.461	Not Supported
Time Management Skills -> Academic Anxiety -> Academic Performance	0.036	0.036	0.022	0.049	1.652	Supported
Student Engagement -> Well-Being -> Academic Performance	0.031	0.030	0.018	0.039	1.761	Supported
Student Engagement -> Academic Anxiety -> Well-Being -> Academic Performance	0.005	0.005	0.004	0.128	1.135	Not Supported
Student Engagement -> Academic Anxiety -> Well-Being	0.028	0.029	0.020	0.086	1.368	Not Supported
Student Engagement -> Academic Anxiety -> Academic Performance	0.033	0.034	0.021	0.057	1.583	Not Supported
Self-Efficacy -> Academic Anxiety -> Well-Being -> Academic Performance	0.007	0.008	0.005	0.079	1.412	Not Supported
Self-Efficacy -> Academic Anxiety -> Well-Being	0.046	0.048	0.024	0.031	1.875	Supported
Self-Efficacy -> Academic Anxiety -> Academic Performance	0.055	0.055	0.024	0.010	2.326	Supported
Parental Support -> Academic Anxiety -> Well-Being -> Academic Performance	0.008	0.008	0.005	0.062	1.543	Not Supported
Parental Support -> Academic Anxiety -> Well-Being	0.048	0.048	0.024	0.024	1.982	Supported
Parental Support -> Academic Anxiety -> Academic Performance	0.057	0.056	0.023	0.007	2.482	Supported
Facilitating Conditions -> Well-Being -> Academic Performance	0.045	0.046	0.024	0.028	1.907	Supported
Academic Anxiety -> Well-Being -> Academic Performance	−0.028	−0.029	0.017	0.050	1.648	Supported

## Data Availability

The data in this study can be provided upon request by sending an e-mail to the corresponding author.

## Supplementary Materials

The following supporting information can be downloaded at: https://www.mdpi.com/article/10.3390/bs14111073/s1 [141,142,143,144,145,146,147].

## References

[1] Improving Postgraduate Students’ Scientific Literacy and Research Ability Using Workshops: Evidence from a Chinese University—Theory, Achievements and Reflection. https://dpi-journals.com/index.php/dtem/article/view/30798.

[2] Friedrich J., Bareis A., Bross M., Bürger Z., Rodríguez Á.C., Effenberger N., Kleinhansl M., Kremer F., Schröder C. (2023). “How Is Your Thesis Going?”—Ph.D. Students’ Perspectives on Mental Health and Stress in Academia. PLoS ONE.

[3] van Rooij E., Fokkens-Bruinsma M., Jansen E. (2021). Factors That Influence PhD Candidates’ Success: The Importance of PhD Project Characteristics. Stud. Contin. Educ..

[4] Council of Graduate Schools (2008). Ph.D. Completion and Attrition: Analysis of Baseline Demographic Data from the Ph.D. Completion Project.

[5] Woolston C., O’Meara S. (2019). PhD Students in China Report Misery and Hope. Nature.

[6] Sarrico C.S. (2022). The Expansion of Doctoral Education and the Changing Nature and Purpose of the Doctorate. High. Educ..

[7] Shen W., Wang C., Oleksiyenko A.V., Zha Q., Chirikov I., Li J. (2024). Historical Trends in PhD Study Abroad and Their Implications for Transforming the Chinese Higher Education System. International Status Anxiety and Higher Education: The Soviet Legacy in China and Russia.

[8] de Oliveira Barbosa M.L., Neves C.E.B. (2020). Internationalization of Higher Education: Institutions and Knowledge Diplomacy. Sociologias.

[9] Nerad M., Cardoso S., Tavares O., Sin C., Carvalho T. (2020). Governmental Innovation Policies, Globalisation, and Change in Doctoral Education Worldwide: Are Doctoral Programmes Converging? Trends and Tensions. Structural and Institutional Transformations in Doctoral Education: Social, Political and Student Expectations.

[10] Zheng G., Cai Y., Oleksiyenko A.V., Zha Q., Chirikov I., Li J. (2024). Collaboration between Europe and China in Doctoral Education: Historical Development and Future Challenges. International Status Anxiety and Higher Education: The Soviet Legacy in China and Russia.

[11] Sökmen Y. (2021). The Role of Self-Efficacy in the Relationship between the Learning Environment and Student Engagement. Educ. Stud..

[12] Wolters C.A., Brady A.C. (2021). College Students’ Time Management: A Self-Regulated Learning Perspective. Educ. Psychol. Rev..

[13] Romano L., Tang X., Hietajärvi L., Salmela-Aro K., Fiorilli C. (2020). Students’ Trait Emotional Intelligence and Perceived Teacher Emotional Support in Preventing Burnout: The Moderating Role of Academic Anxiety. Int. J. Environ. Res. Public Health.

[14] Ling X., Chen J., Chow D.H.K., Xu W., Li Y. (2022). The “Trade-Off” of Student Well-Being and Academic Achievement: A Perspective of Multidimensional Student Well-Being. Front. Psychol..

[15] Romano L., Angelini G., Consiglio P., Fiorilli C. (2021). Academic Resilience and Engagement in High School Students: The Mediating Role of Perceived Teacher Emotional Support. Eur. J. Investig. Health Psychol. Educ..

[16] Salazar C. (2024). “I Knew It Was Gonna Be Hard, but I Always Knew I Had Support from My Parents”: The Role of Family on Undocumented Students’ College Aspirations and Persistence. J. Coll. Stud. Retent. Res. Theory Pract..

[17] Tomaszewski W., Xiang N., Western M. (2020). Student Engagement as a Mediator of the Effects of Socio-Economic Status on Academic Performance among Secondary School Students in Australia. Br. Educ. Res. J..

[18] Al-Rahmi A.M., Al-Rahmi W.M., Alturki U., Aldraiweesh A., Almutairy S., Al-Adwan A.S. (2021). Exploring the Factors Affecting Mobile Learning for Sustainability in Higher Education. Sustainability.

[19] Gisemba Bagaka’s J., Bransteter I., Rispinto S., Badillo N. (2015). Exploring Student Success in a Doctoral Program: The Power of Mentorship and Research Engagement. Int. J. Dr. Stud..

[20] Razak A.A., Ramdan M.R., Mahjom N., Zabit M.N.M., Muhammad F., Hussin M.Y.M., Abdullah N.L. (2022). Improving Critical Thinking Skills in Teaching through Problem-Based Learning for Students: A Scoping Review. Int. J. Learn. Teach. Educ. Res..

[21] Stewart-Wells A.G., Keenan K.M. (2020). Assessing Doctoral Students: A Background on Comprehensive and Authentic Assessments. J. Contin. High. Educ..

[22] Lorenzetti D.L., Nowell L., Jacobsen M., Lorenzetti L., Clancy T., Freeman G., Oddone Paolucci E. (2020). The Role of Peer Mentors in Promoting Knowledge and Skills Development in Graduate Education. Educ. Res. Int..

[23] An Le D.T.B., Hockey J. (2022). Critical Thinking in the Higher Education Classroom: Knowledge, Power, Control and Identities. Br. J. Sociol. Educ..

[24] Hewett I. (2021). The Vanishing Discipline: The Threat to Musicology. Search J. New Music Cult..

[25] Hardegger D. (2020). The Rise of the Modern PhD: PhD Candidates at the University of Berlin and at Columbia University, New York, from 1871 to 1913.

[26] Li J., Xue E. (2022). Exploring High-Quality Institutional Internationalization for Higher Education Sustainability in China: Evidence from Stakeholders. Sustainability.

[27] Dong M., Xiao Y., Shi C., Zeng W., Wu F., Li G. (2022). Which Contributes to Clinical Performance: Academic Output or Person–Environment Fit?. Front. Public Health.

[28] Cardoso S., Santos S., Diogo S., Soares D., Carvalho T. (2022). The Transformation of Doctoral Education: A Systematic Literature Review. High. Educ..

[29] Pyhältö K., Peltonen J., Castelló M., McAlpine L. (2020). What Sustains Doctoral Students’ Interest? Comparison of Finnish, UK and Spanish Doctoral Students’ Perceptions. Comp. J. Comp. Int. Educ..

[30] Sverdlik A., Hall N.C., McAlpine L., Hubbard K. (2018). The PhD Experience: A Review of the Factors Influencing Doctoral Students’ Completion, Achievement, and Well-Being. Int. J. Dr. Stud..

[31] Richards K.A.R., Shiver V.N. (2020). Managing the Critical Friendship: Using Self-Study in the Doctoral Supervision Process. Stud. Teach. Educ..

[32] Gardner S.K., Mendoza P. (2023). On Becoming a Scholar: Socialization and Development in Doctoral Education.

[33] Vähämäki M., Saru E., Palmunen L.-M. (2021). Doctoral Supervision as an Academic Practice and Leader–Member Relationship: A Critical Approach to Relationship Dynamics. Int. J. Manag. Educ..

[34] Bandura A. (1977). Self-efficacy: Toward a unifying theory of behavioral change. Psychol. Rev..

[35] Al-Abyadh M.H.A., Abdel Azeem H.A.H. (2022). Academic Achievement: Influences of University Students’ Self-Management and Perceived Self-Efficacy. J. Intell..

[36] Matheka H., Jansen E., Hofman A. (2020). Kenyan Doctoral Students’ Success: Roles of Motivation and Self-Efficacy. Perspect. Educ..

[37] Hemmings B., Kay R. (2016). The Relationship between Research Self-Efficacy, Research Disposition and Publication Output. Educ. Psychol..

[38] Sverdlik A., Hall N.C. (2020). Not Just a Phase: Exploring the Role of Program Stage on Well-Being and Motivation in Doctoral Students. J. Adult Contin. Educ..

[39] Lachance K., Heustis R.J., Loparo J.J., Venkatesh M.J. (2020). Self-Efficacy and Performance of Research Skills among First-Semester Bioscience Doctoral Students. CBE Life Sci. Educ..

[40] Zarza-Alzugaray F.J., Casanova O., McPherson G.E., Orejudo S. (2020). Music Self-Efficacy for Performance: An Explanatory Model Based on Social Support. Front. Psychol..

[41] Jiang J. (2024). Impact of Music Learning on Students’ Psychological Development with Mediating Role of Self-Efficacy and Self-Esteem. PLoS ONE.

[42] Guo H., Zhou Z., Ma F., Chen X. (2024). Doctoral Students’ Academic Performance: The Mediating Role of Academic Motivation, Academic Buoyancy, and Academic Self-Efficacy. Heliyon.

[43] Acoba E.F. (2024). Social Support and Mental Health: The Mediating Role of Perceived Stress. Front. Psychol..

[44] Lerang M.S., Ertesvåg S.K., Virtanen T. (2021). Patterns of Teachers’ Instructional Support Quality and the Association with Job Satisfaction and Collegial Collaboration. Educ. Psychol..

[45] Posselt J. (2018). Normalizing Struggle: Dimensions of Faculty Support for Doctoral Students and Implications for Persistence and Well-Being. J. High. Educ..

[46] Li Z., Huang J., Hussain S., Shu T. (2023). How Do Anxiety and Stress Impact the Performance of Chinese Doctoral Students through Self-Regulated Learning?—A Multi-Group Analysis. Front. Psychol..

[47] Barrett M.S., Creech A., Zhukov K. (2021). Creative Collaboration and Collaborative Creativity: A Systematic Literature Review. Front. Psychol..

[48] Zhang H., Liu L., Li X., Sun Y. (2024). How Doctoral Students Understand Academic Identity in China: A Qualitative Study Based on the Grounded Theory. Educ. Sci..

[49] Piskorz-Ryń O., Chikwe C. (2024). Parental Involvement and Its Influence on Academic Achievement. Iran. J. Educ. Sociol..

[50] Ma Y., Yu A., Ma H., Zhao Y., Liu X., Zhai H., Gao Y. (2024). A Narrative Review of Anxiety Regulation in PhD Students Based on Green Model. Front. Psychol..

[51] Borjas M.-P., Ricardo C., Escalante-Barrios E.L., Valencia J., Aparicio J. (2020). Financial Independence and Academic Achievement: Are There Key Factors of Transition to Adulthood for Young Higher Education Students in Colombia?. Front. Psychol..

[52] Hwang W., Jung E. (2021). Parenting Practices, Parent–Child Relationship, and Perceived Academic Control in College Students. J. Adult Dev..

[53] Xu T., Zuo F., Zheng K. (2024). Parental Educational Expectations, Academic Pressure, and Adolescent Mental Health: An Empirical Study Based on CEPS Survey Data. Int. J. Ment. Health Promot..

[54] Lourenço A.A., Paiva M.O. (2024). Academic Performance of Excellence: The Impact of Self-Regulated Learning and Academic Time Management Planning. Knowledge.

[55] Wilson R., Joiner K., Abbasi A. (2021). Improving Students’ Performance with Time Management Skills. J. Univ. Teach. Learn. Pract..

[56] Cho K.W., Serrano D.M. (2020). Noncognitive Predictors of Academic Achievement Among Nontraditional and Traditional Ethnically Diverse College Students. J. Contin. High. Educ..

[57] Aeon B., Faber A., Panaccio A. (2021). Does Time Management Work? A Meta-Analysis. PLoS ONE.

[58] Jääskeläinen T. (2022). Music Students’ Workload, Stress, and Coping in Higher Education: Evidence-Based Policymaking. Front. Psychol..

[59] Cabellos B., Siddiq F., Scherer R. (2024). The Moderating Role of School Facilitating Conditions and Attitudes towards ICT on Teachers’ ICT Use and Emphasis on Developing Students’ Digital Skills. Comput. Hum. Behav..

[60] Batucan G.B., Gonzales G.G., Balbuena M.G., Pasaol K.R.B., Seno D.N., Gonzales R.R. (2022). An Extended UTAUT Model to Explain Factors Affecting Online Learning System Amidst COVID-19 Pandemic: The Case of a Developing Economy. Front. Artif. Intell..

[61] Adeshina K. (2021). Collection Management and Utilization of E-Resources by Post Graduate Student in University of Ibadan, Nigeria. Libr. Philos. Pract..

[62] Moirano R., Sánchez M.A., Štěpánek L. (2020). Creative Interdisciplinary Collaboration: A Systematic Literature Review. Think. Ski. Creat..

[63] Sartas M., Schut M., Proietti C., Thiele G., Leeuwis C. (2020). Scaling Readiness: Science and Practice of an Approach to Enhance Impact of Research for Development. Agric. Syst..

[64] Pace I. (2016). Composition and performance can be, and often have been, research. Tempo.

[65] Heilporn G., Lakhal S., Bélisle M. (2021). An Examination of Teachers’ Strategies to Foster Student Engagement in Blended Learning in Higher Education. Int. J. Educ. Technol. High. Educ..

[66] Astin A.W. (1984). Student Involvement: A Developmental Theory for Higher Education. J. Coll. Stud. Pers..

[67] Wang M., Eccles J.S. (2012). Adolescent Behavioral, Emotional, and Cognitive Engagement Trajectories in School and Their Differential Relations to Educational Success. J. Res. Adolesc..

[68] So J.C.-H., Ho Y.H., Wong A.K.-L., Chan H.C.B., Tsang K.H.-Y., Chan A.P.-L., Wong S.C.-W. (2023). Analytic Study for Predictor Development on Student Participation in Generic Competence Development Activities Based on Academic Performance. IEEE Trans. Learn. Technol..

[69] Trolian T.L., Archibald G.C., Jach E.A. (2022). Well-Being and Student–Faculty Interactions in Higher Education. High. Educ. Res. Dev..

[70] Zhao Y., Zheng Z., Pan C., Lulu Z. (2021). Self-Esteem and Academic Engagement Among Adolescents: A Moderated Mediation Model. Front. Psychol..

[71] Chen J., Zhao Z. (2024). A Study on the Influence of Academic Passion on PhD Students’ Research Engagement—The Role of Ambidextrous Learning and Academic Climate. PLoS ONE.

[72] Wang F., Huang X., Zeb S., Liu D., Wang Y. (2022). Impact of Music Education on Mental Health of Higher Education Students: Moderating Role of Emotional Intelligence. Front. Psychol..

[73] Hsu J.L., Goldsmith G.R. (2021). Instructor Strategies to Alleviate Stress and Anxiety among College and University STEM Students. CBE Life Sci. Educ..

[74] Robinson O.J., Vytal K., Cornwell B.R., Grillon C. (2013). The Impact of Anxiety upon Cognition: Perspectives from Human Threat of Shock Studies. Front. Hum. Neurosci..

[75] Ojo A., Oginni O.G., Akinrinola O.E., Oginni R.I. (2023). Impact of Cognitive-Behavioral Intervention on Alleviating Depression and Anxiety in Mathematics: Enhancing Students’ Learning Experience and Academic Performance. Voice Publ..

[76] Aprilia A., Aminatun D. (2022). Investigating memory loss: How depression affects students’ memory endurance. J. Engl. Lang. Teach. Learn..

[77] Calimag M.M.P. (2021). Surfacing Anger and Anxiety in Graduate Research Writing: A Prose Poetic Journey. J. Med. Univ. St. Tomas.

[78] Zhu P., Xu T., Xu H., Ji Q., Wang W., Qian M., Shi G. (2023). Relationship between Anxiety, Depression and Learning Burnout of Nursing Undergraduates after the COVID-19 Epidemic: The Mediating Role of Academic Self-Efficacy. Int. J. Environ. Res. Public Health.

[79] Wang Q., Yang R. (2024). The Influence of Music Performance Anxiety on Career Expectations of Early Musical Career Students: Self-Efficacy as a Moderator. Front. Psychol..

[80] Lorber M., Černe Kolarič J., Kmetec S., Kegl B. (2023). Association between Loneliness, Well-Being, and Life Satisfaction before and during the COVID-19 Pandemic: A Cross-Sectional Study. Sustainability.

[81] Oishi S., Diener E., Lucas R.E., Snyder C.R., Lopez S.J., Edwards L.M., Marques S.C. (2021). Subjective Well-Being: The Science of Happiness and Life Satisfaction. The Oxford Handbook of Positive Psychology.

[82] Donohue D.K., Bornman J. (2021). Academic Well-Being in Higher Education: A Cross-Country Analysis of the Relationship Between Perceptions of Instruction and Academic Well-Being. Front. Psychol..

[83] Liu C., Xie Y., Xu Y., Song Z., Tang J., Shen J., Jiang Z., Shen C., Zhan X., Zheng C. (2024). Assessing the Stress-Relief Impact of an Art-Based Intervention Inspired by the Broaden-and-Build Theory in College Students. Front. Psychol..

[84] Collie R.J. (2022). Instructional Support, Perceived Social-Emotional Competence, and Students’ Behavioral and Emotional Well-Being Outcomes. Educ. Psychol..

[85] Sun J. (2022). Exploring the Impact of Music Education on the Psychological and Academic Outcomes of Students: Mediating Role of Self-Efficacy and Self-Esteem. Front. Psychol..

[86] Dingle G.A., Sharman L.S., Bauer Z., Beckman E., Broughton M., Bunzli E., Davidson R., Draper G., Fairley S., Farrell C. (2021). How Do Music Activities Affect Health and Well-Being? A Scoping Review of Studies Examining Psychosocial Mechanisms. Front. Psychol..

[87] Hair J., Hair J.F., Sarstedt M., Ringle C.M., Gudergan S.P. (2023). Advanced Issues in Partial Least Squares Structural Equation Modeling.

[88] Shmueli G., Sarstedt M., Hair J.F., Cheah J.-H., Ting H., Vaithilingam S., Ringle C.M. (2019). Predictive Model Assessment in PLS-SEM: Guidelines for Using PLSpredict. Eur. J. Mark..

[89] Manley S.C., Williams R.I., Hair J.F. (2024). Enhancing TQM’s Effect on Small Business Performance: A PLS-SEM Exploratory Study of TQM Applied with a Comprehensive Strategic Approach. TQM J..

[90] Memon M.A., Ramayah T., Cheah J.H., Ting H., Chuah F., Cham T.H. (2021). PLS-SEM Statistical Programs: A Review. J. Appl. Struct. Equ. Model..

[91] Hair J.F., Howard M.C., Nitzl C. (2020). Assessing Measurement Model Quality in PLS-SEM Using Confirmatory Composite Analysis. J. Bus. Res..

[92] Stauropoulou A., Sardianou E., Malindretos G., Evangelinos K., Nikolaou I. (2023). The Role of Customers’ Awareness towards the Sustainable Development Goals (SDGs) of Banks on Their Behavior. Env. Sci. Pollut. Res. Int..

[93] Yuan J., Wang S., Pan C. (2022). Mechanism of Impact of Big Data Resources on Medical Collaborative Networks from the Perspective of Transaction Efficiency of Medical Services: Survey Study. J. Med. Internet Res..

[94] Rombach M., Dean D.L., Baird T., Kambuta J. (2022). Should I Pay or Should I Grow? Factors Which Influenced the Preferences of US Consumers for Fruit, Vegetables, Wine and Beer during the COVID-19 Pandemic. Foods.

[95] Queiroz M.M., Fosso Wamba S., Chiappetta Jabbour C.J., Machado M.C. (2022). Supply Chain Resilience in the UK during the Coronavirus Pandemic: A Resource Orchestration Perspective. Int. J. Prod. Econ..

[96] Alsisi E.A., Al-Ashaab A., Abualfaraa W.A. (2020). The Development of a Smart Health Awareness Message Framework Based on the Use of Social Media: Quantitative Study. J. Med. Internet Res..

[97] George A.K., Singh M., Pushpakumar S., Homme R.P., Hardin S.J., Tyagi S.C. (2020). Dysbiotic 1-Carbon Metabolism in Cardiac Muscle Remodeling. J. Cell Physiol..

[98] Iskandar Y.H.P., Subramaniam G., Majid M.I.A., Ariff A.M., Rao G.K.L. (2020). Predicting Healthcare Professionals’ Intention to Use Poison Information System in a Malaysian Public Hospital. Health Inf. Sci. Syst..

[99] Wijaya T.T., Cao Y., Weinhandl R., Yusron E., Lavicza Z. (2022). Applying the UTAUT Model to Understand Factors Affecting Micro-Lecture Usage by Mathematics Teachers in China. Mathematics.

[100] Sarabia-Andreu F., Sarabia-Sánchez F.J., Parra-Meroño M.C., Moreno-Albaladejo P. (2020). A Multifaceted Explanation of the Predisposition to Buy Organic Food. Foods.

[101] Tan B.C., Pang S.M., Lau T.C. (2022). Marketing Organic Food from Millennials’ Perspective: A Multi-Theoretical Approach. Foods.

[102] Ahadzadeh A.S., Wu S.L., Ong F.S., Deng R. (2021). The Mediating Influence of the Unified Theory of Acceptance and Use of Technology on the Relationship Between Internal Health Locus of Control and Mobile Health Adoption: Cross-Sectional Study. J. Med. Internet Res..

[103] Lu D., Qiu S., Xian D., Zhang J., Zhang Y., Liu X., Yang W., Liu X. (2022). Psychotic-like Experiences and Associated Socio-Demographic Factors among Pregnant Women in Each Trimester in China. Front. Psychiatry.

[104] Zhan D., Zhang Q., Kwan M.-P., Liu J., Zhan B., Zhang W. (2022). Impact of Urban Green Space on Self-Rated Health: Evidence from Beijing. Front. Public Health.

[105] Bandura A., Locke E.A. (2003). Negative Self-Efficacy and Goal Effects Revisited. J. Appl. Psychol..

[106] Khan M. (2023). Academic Self-Efficacy, Coping, and Academic Performance in College. Int. J. Undergrad. Res. Creat. Act..

[107] Brandt L., Liu S., Heim C., Heinz A. (2022). The Effects of Social Isolation Stress and Discrimination on Mental Health. Transl. Psychiatry.

[108] Freire C., Ferradás M.d.M., Regueiro B., Rodríguez S., Valle A., Núñez J.C. (2020). Coping Strategies and Self-Efficacy in University Students: A Person-Centered Approach. Front. Psychol..

[109] Putwain D.W., Wood P., Pekrun R. (2022). Achievement Emotions and Academic Achievement: Reciprocal Relations and the Moderating Influence of Academic Buoyancy. J. Educ. Psychol..

[110] Ward R.T., Lotfi S., Sallmann H., Lee H.-J., Larson C.L. (2020). State Anxiety Reduces Working Memory Capacity but Does Not Impact Filtering Cost for Neutral Distracters. Psychophysiology.

[111] Cao F., Zhang L., Li M., Xie Z. (2024). Subjective Well-Being among PhD Students in Mainland China: The Roles of Psychological Capital and Academic Engagement. Front. Psychol..

[112] Henning G., Bjälkebring P., Stenling A., Thorvaldsson V., Johansson B., Lindwall M. (2019). Changes in Within- and between-Person Associations between Basic Psychological Need Satisfaction and Well-Being after Retirement. J. Res. Personal..

[113] Patall E.A., Kennedy A.A.U., Yates N., Zambrano J., Lee D., Vite A. (2022). The Relations between Urban High School Science Students’ Agentic Mindset, Agentic Engagement, and Perceived Teacher Autonomy Support and Control. Contemp. Educ. Psychol..

[114] Al Makhamreh M., Stockley D. (2020). Mentorship and Well-Being. Int. J. Mentor. Coach. Educ..

[115] Choi Y.H., Bouwma-Gearhart J., Ermis G. (2021). Doctoral Students’ Identity Development as Scholars in the Education Sciences: Literature Review and Implications. Int. J. Dr. Stud..

[116] Yu J., Huang C., He T., Wang X., Zhang L. (2022). Investigating Students’ Emotional Self-Efficacy Profiles and Their Relations to Self-Regulation, Motivation, and Academic Performance in Online Learning Contexts: A Person-Centered Approach. Educ. Inf. Technol..

[117] Afzal A., Rafiq S., Kanwal A. (2023). The Influence of Teacher-Student Relationships on Students’ Academic Achievement at University Level. Gomal Univ. J. Res..

[118] Cutrín O., Maneiro L., Chowdhury Y., Kulis S.S., Marsiglia F.F., Gómez Fraguela J.A. (2022). Longitudinal Associations between Parental Support and Parental Knowledge on Behavioral and Emotional Problems in Adolescents. J. Youth Adolesc..

[119] Rodriguez-Oramas A., Morla-Folch T., Vieites Casado M., Ruiz-Eugenio L. (2022). Improving Students’ Academic Performance and Reducing Conflicts through Family Involvement in Primary School Learning Activities: A Mexican Case Study. Camb. J. Educ..

[120] Elsayed H., O’Connor C., Leyritana K., Salvana E., Cox S.E. (2021). Depression, Nutrition, and Adherence to Antiretroviral Therapy in Men Who Have Sex with Men in Manila, Philippines. Front. Public Health.

[121] Tan C.Y., Lyu M., Peng B. (2020). Academic Benefits from Parental Involvement Are Stratified by Parental Socioeconomic Status: A Meta-Analysis. Parenting.

[122] Zeng Q., Liang Z., Zhang M., Xia Y., Li J., Kang D., Yi D., Wang J. (2021). Impact of Academic Support on Anxiety and Depression of Chinese Graduate Students During the COVID-19 Pandemic: Mediating Role of Academic Performance. Psychol. Res. Behav. Manag..

[123] Sun Y., Lin S.-Y., Chung K.K.H. (2020). University Students’ Perceived Peer Support and Experienced Depressive Symptoms during the COVID-19 Pandemic: The Mediating Role of Emotional Well-Being. Int. J. Environ. Res. Public Health.

[124] Tsitsia B., Afenu D., Kabbah S., Attigah A., Bimpeh G. (2021). Effective Time Management Practices Among Colleges of Education Students. J. Hum. Resour. Leadersh..

[125] Ariastuti M.D., Wahyudin A.Y. (2022). Exploring Academic Performance and Learning Style of Undergraduate Students in English Education Program. J. Engl. Lang. Teach. Learn..

[126] Pravita A.R., Kuswandono P. (2022). Writing Anxiety and Academic Procrastination on Undergraduate Thesis Writing: The Role of Self-Regulation. J. Engl. Educ. Linguist. Stud..

[127] Deb S., Thomas S., Bose A., Aswathi T. (2020). Happiness, Meaning, and Satisfaction in Life as Perceived by Indian University Students and Their Association with Spirituality. J. Relig. Health.

[128] Realyvásquez-Vargas A., Maldonado-Macías A.A., Arredondo-Soto K.C., Baez-Lopez Y., Carrillo-Gutiérrez T., Hernández-Escobedo G. (2020). The Impact of Environmental Factors on Academic Performance of University Students Taking Online Classes during the COVID-19 Pandemic in Mexico. Sustainability.

[129] Ngui G.K., Lay Y.F. (2020). The Effect of Emotional Intelligence, Self-Efficacy, Subjective Well-Being and Resilience on Student Teachers’ Perceived Practicum Stress: A Malaysian Case Study. Eur. J. Educ. Res..

[130] Pappa S., Elomaa M., Perälä-Littunen S. (2020). Sources of Stress and Scholarly Identity: The Case of International Doctoral Students of Education in Finland. High. Educ..

[131] Adams A.-M., Wilson H., Money J., Palmer-Conn S., Fearn J. (2020). Student Engagement with Feedback and Attainment: The Role of Academic Self-Efficacy. Assess. Eval. High. Educ..

[132] Hidayat R., Moosavi Z., Hadisaputra P. (2022). Achievement Goals, Well-Being and Lifelong Learning: A Mediational Analysis. Int. J. Instr..

[133] Knifsend C.A. (2020). Intensity of Activity Involvement and Psychosocial Well-Being among Students. Act. Learn. High. Educ..

[134] Kim H.J., Yi P., Hong J.I. (2020). Students’ Academic Use of Mobile Technology and Higher-Order Thinking Skills: The Role of Active Engagement. Educ. Sci..

[135] Tan C.-Y., Chuah C.-Q., Lee S.-T., Tan C.-S. (2021). Being Creative Makes You Happier: The Positive Effect of Creativity on Subjective Well-Being. Int. J. Environ. Res. Public Health.

[136] Caviola S., Toffalini E., Giofrè D., Ruiz J.M., Szűcs D., Mammarella I.C. (2022). Math Performance and Academic Anxiety Forms, from Sociodemographic to Cognitive Aspects: A Meta-Analysis on 906,311 Participants. Educ. Psychol. Rev..

[137] Wu Y., Yin G., Zhang Y. (2022). Experience and Perceptions of Chinese University Students Regarding the COVID-19 Pandemic: A Qualitative Analysis. Front. Public Health.

[138] Faisal R.A., Jobe M.C., Ahmed O., Sharker T. (2022). Mental Health Status, Anxiety, and Depression Levels of Bangladeshi University Students During the COVID-19 Pandemic. Int. J. Ment. Health Addict..

[139] Morales-Rodríguez F.M., Espigares-López I., Brown T., Pérez-Mármol J.M. (2020). The Relationship between Psychological Well-Being and Psychosocial Factors in University Students. Int. J. Environ. Res. Public Health.

[140] Crowley C., Kapitula L.R., Munk D. (2022). Mindfulness, Happiness, and Anxiety in a Sample of College Students before and after Taking a Meditation Course. J. Am. Coll. Health.

[141] Greco A., Annovazzi C., Palena N., Camussi E., Rossi G., Steca P. (2022). Self-Efficacy Beliefs of University Students: Examining Factor Validity and Measurement Invariance of the New Academic Self-Efficacy Scale. Front. Psychol..

[142] Rooney J.A., Gottlieb B.H. (2007). Development and Initial Validation of a Measure of Supportive and Unsupportive Managerial Behaviors. J. Vocat. Behav..

[143] Mooney P., Epstein M.H., Ryser G., Pierce C.D. (2005). Reliability and Validity of the Behavioral and Emotional Rating Scale-Second Edition: Parent Rating Scale. Child. Sch..

[144] Tohan M.M., Ahmed F., Juie I.J., Kabir A., Howlader M.H., Rahman M.A. (2024). Knowledge Attitude and Convenience on Self-Medication Practices among University Students in Bangladesh Exploration Using Structural Equation Modeling Approach. Sci. Rep..

[145] Spitzer R.L., Kroenke K., Williams J.B.W., Löwe B. (2006). A Brief Measure for Assessing Generalized Anxiety Disorder: The GAD-7. Arch. Intern. Med..

[146] Blasco-Belled A., Alsinet C. (2022). The Architecture of Psychological Well-Being: A Network Analysis Study of the Ryff Psychological Well-Being Scale. Scand. J. Psychol..

[147] Ward A.M., Brennan N.M. (2020). Developing a Student-Doctoral Education Fit Analytical Model to Assess Performance. Stud. High. Educ..

