# Supplementary material for: The Influence of Multiple Factors on Musicology Doctoral Students’ Academic Performance: An Empirical Study Based in China
Source: Behav Sci (Basel). 2024 Nov 11;14(11):1073. doi: 10.3390/bs14111073 (PMC11591338; doi:10.3390/bs14111073)
Supplement: Supplementary file 1 [file behavsci-14-01073-s001.zip › behavsci-3239022-supplementary.pdf]

Supplementary Materials

Detailed questionnaires

Variable	Code	English version	Chinese version	References
**Self-Efficacy**	SE1: Satisfying requirements	I am confident in my ability to complete my doctoral thesis on time and meet all academic requirements.	我有信心按时完成博士论文并满足所有学术要求。	[1]
	SE2: Excellent outcomes	Despite facing challenges, I am optimistic about achieving excellent academic outcomes.	即使面对挑战，我相信自己能取得优异的学术成绩。	
	SE3: Skilled presentations	I am skilled in effectively presenting and elucidating research findings at academic conferences.	我有能力在学术会议上有效展示和解释研究成果。	
	SE4: Preparedness	I feel well-prepared to tackle a variety of complex issues in my academic research.	我有信心应对学术研究中的各种复杂问题。	
**Teacher Support**	TS1: Clear standards	My advisor has established clear expectations and academic standards for my research.	我的导师为我的研究设定了明确的期望和学术标准。	[2]
	TS2: Prompt response	My supervisor responds promptly to any inquiries I have during the research process.	我的导师能及时回应我在研究过程中遇到的问题。	
	TS3: Equal treating	My advisor treats all students with impartiality.	我的导师对所有学生一视同仁。	
	TS4: Supporting decisions	My advisor supports and validates my academic decisions and choices.	我的导师支持并认可我的学术决策和选择。	
	TS5: Promoting collaborations	My advisor promotes collaboration among students to accomplish academic tasks together.	我的导师鼓励学生积极合作，共同完成学术任务。	
**Parental Support**	PS1: Financial support	My parents provided ample financial support throughout my PhD studies.	我的父母在我攻读博士期间提供了足够的经济支持。	[3]
	PS2: Understanding and encouraging	My parents have been understanding and encouraging me to excel in my academic and career endeavors.	我的父母理解并激励我在学术选择和职业发展规划中追求卓越。	
	PS3: Emotional support	During periods of academic stress, my parents have offered emotional comfort and support.	我的父母在我面临学术压力时给予了情感上的安慰和支持。	

**Time Management Skills**	TM1: Prioritizing tasks	I am adept at setting clear priorities for my academic tasks.	我能够为我的学术任务设定清晰的优先级。	[4]
	TM2: Strict schedules	I develop detailed schedules to organize my academic tasks and rigorously follow these plans.	我会制定详细的日程表来规划学术任务，并严格遵循这些计划。	
	TM3: Downtime management	When I experience downtime in my academic work, I efficiently utilize this time to advance other research-related tasks.	当我在学术工作中遇到等待时间时，会有效利用这些时间完成其他研究相关任务。	
	TM4: Effective multitasking	Even when juggling multiple tasks, I effectively manage and allocate my time.	即使面对多个任务，我仍能有效掌控和分配时间。	
**Facilitating Conditions**	FC1: Helpful people	Whenever I need assistance, I consistently find someone willing to help.	在我需要帮助时，我总能找到愿意提供帮助的人。	[5]
	FC2: Access to resources	I can easily access all the literature, data, and resources required for my academic research.	我能够方便地获取完成学术研究所需的文献、数据和其他资源。	
	FC3: Workshops and training sessions	The workshops and training sessions I have attended have significantly enhanced my academic skills.	我参加的培训和研讨会对提升我的学术技能有显著帮助。	
**Student Engagement**	SEng1: Intense focus	I have been intensely focused on my PhD research.	我非常专注于我的博士研究。	[6]
	SEng2: Making best effort	I believe I have exerted maximum effort throughout my PhD studies.	我认为自己在博士学习期间尽了最大的努力。	
	SEng3: No withdrawal intention	I have no intention of withdrawing from my doctoral program.	我没有计划从博士教育中辍学。	
	SEng4: Great satisfaction	Overall, I find great satisfaction in my current PhD studies.	总的来说，我很享受目前的博士课程。	
**Academic Anxiety**	AA1: Experiencing anxiety	In the past two weeks, I have often experienced feelings of nervousness, unease, or academic stress.	过去两周内，我常常感到紧张、不安或对学术研究感到压力。	[7]
	AA2: Worries about progress	In the past two weeks, I have been unable to stop or control my concerns about the progress of my PhD research.	过去两周内，我无法停止或控制对博士研究进展的担忧。	
	AA3: Excessive worries about outcomes	In the past two weeks, I have been overly anxious about academic tasks or research outcomes.	过去两周内，我过度担心学术任务或研究结果。	
	AA4: Difficult to relax	In the past two weeks, I have found it challenging to unwind	过去两周内，我发现自己难以在繁重的学术任务中放松。	

		from the demands of a heavy academic workload.		
	AA5: Significant unease	In the past two weeks, my PhD research has recently caused significant unease, making it difficult to concentrate.	过去两周内，博士研究让我感到非常不安，难以专注。	
	AA6: Feeling irritable or frustrated	In the past two weeks, in the face of academic challenges, I have occasionally felt irritable or frustrated.	过去两周内，在应对学术挑战时，我容易感到烦躁或恼怒。	
**Well-Being**	WB1: Pleased with progress	Overall, I am pleased with the progress I have made in my academic research and professional development.	总体来说，我对自己在学术研究和职业发展中的进展感到满意。	[8]
	WB2: Growing in research and challenges	I have continuously grown in my academic research and successfully navigated various challenges.	我对自己的学术生涯有明确的方向感和目标。	
	WB3: Meeting expectations	I have met or exceeded the expected performance levels in all my PhD courses.	我在学术研究中不断成长，并能够克服遇到的各种挑战。	
**Academic Performance**	AP1: Improving academic skills	Throughout my doctoral studies, my academic skills have significantly improved.	我在博士期间的每门课程中都达到了预期的成绩水平。	[9]
	AP2: Advancing writing skills	My academic writing skills improved significantly during my doctoral studies.	在博士研究过程中，我的学术写作技能显著提升。	
	AP3: Confident showcasing	During my PhD, I confidently presented research findings at both national and international academic conferences.	博士期间，我能自信地在国内外学术会议上展示研究成果。	
	AP4: Advancing critical thinking abilities	My critical thinking abilities have notably advanced during my PhD studies.	博士阶段，我的批判性思维能力明显进步。	
	AP5: Acquiring knowledge	The knowledge I acquired during my PhD is highly applicable to my future career.	我在博士学习期间获取了对未来职业有用的知识。	

References

1. Greco, A.; Annovazzi, C.; Palena, N.; Camussi, E.; Rossi, G.; Steca, P. Self-Efficacy Beliefs of University Students: Examining Factor Validity and Measurement Invariance of the New Academic Self-Efficacy Scale. *Front. Psychol.* **2022**, *12*. https://doi.org/10.3389/fpsyg.2021.498824.
2. Rooney, J.A.; Gottlieb, B.H. Development and Initial Validation of a Measure of Supportive and Unsupportive Managerial Behaviors. *J. Vocat. Behav.* **2007**, *71*, 186–203. https://doi.org/10.1016/j.jvb.2007.03.006.
3. Mooney, P.; Epstein, M.H.; Ryser, G.; Pierce, C.D. Reliability and Validity of the Behavioral and Emotional Rating Scale-Second Edition: Parent Rating Scale. *Child. Sch.* **2005**, *27*, 147–155. https://doi.org/10.1093/cs/27.3.147.
4. Wolters, C.A.; Brady, A.C. College Students' Time Management: A Self-Regulated Learning Perspective. *Educ. Psychol. Rev.* **2021**, *33*, 1319–1351. https://doi.org/10.1007/s10648-020-09519-z.
5. Tohan, M.M.; Ahmed, F.; Juie, I.J.; Kabir, A.; Howlader, M.H.; Rahman, M.A. Knowledge Attitude and Convenience on Self-Medication Practices among University Students in Bangladesh Exploration Using Structural Equation Modeling Approach. *Sci. Rep.* **2024**, *14*, 10837. https://doi.org/10.1038/s41598-024-60931-9.

6. Sverdlik, A.; Hall, N. C.; McAlpine, L.; Hubbard, K. The PhD Experience: A Review of the Factors Influencing Doctoral Students' Completion, Achievement, and Well-Being. *International Journal of Doctoral Studies* **2018**, *13*, 361–388.
7. Spitzer, R.L.; Kroenke, K.; Williams, J.B.W.; Löwe, B. A Brief Measure for Assessing Generalized Anxiety Disorder: The GAD-7. *Arch. Intern. Med.* **2006**, *166*, 1092–1097. https://doi.org/10.1001/archinte.166.10.1092.
8. Blasco-Belled, A.; Alsinet, C. The Architecture of Psychological Well-Being: A Network Analysis Study of the Ryff Psychological Well-Being Scale. *Scand. J. Psychol.* **2022**, *63*, 199–207. https://doi.org/10.1111/sjop.12795.
9. Ward, A.M.; Brennan, N.M. Developing a Student-Doctoral Education Fit Analytical Model to Assess Performance. *Stud. High. Educ.* **2020**, *45*, 1448–1460. https://doi.org/10.1080/03075079.2018.1545758.
